# A novel free-air diesel and ozone enrichment (FADOE) research platform

**DOI:** 10.1016/j.mex.2024.102635

**Published:** 2024-02-27

**Authors:** Adedayo O. Mofikoya, Laura James, Neil J. Mullinger, James M.W. Ryalls, Robbie D. Girling

**Affiliations:** a School of Agriculture, Policy and Development, University of Reading, Whiteknights, Earley Gate, Reading RG6 6EU, UK; b School of Geography, Earth and Environmental Sciences, University of Birmingham, Edgbaston, Birmingham B15 2TT, UK; c UK Centre for Ecology & Hydrology, Penicuik, Midlothian EH26 0QB, UK; d Centre for Sustainable Agricultural Systems, Institute for Life Sciences and the Environment, University of Southern Queensland, Toowoomba, Queensland 4350, Australia

**Keywords:** Free-Air Diesel and Ozone Exposure (FADOE), Air pollution, Diesel exhaust, Global change ecology, Tropospheric ozone

## Abstract

Air pollution is an escalating concern in the modern world, posing substantial threats to ecosystem processes. While the importance of comprehending the impact of pollutants on natural environments is evident, conducting rigorous field-based experiments presents formidable challenges. Elevating pollutant concentrations within open air environments in a controlled manner is complex. Nonetheless, such real-world experiments are invaluable for revealing the genuine influence of air pollutants on ecosystems and their functioning. Field-scale measurements have emerged as a pivotal avenue for advancing our understanding of the interactions between air pollutants and the natural world, providing unique insights into ecosystem dynamics, including critical processes like pollination and natural pest regulation. In atmospheric and ecological research, free-air exposure systems have proven effective in elevating carbon dioxide (CO_2_) and ozone (O_3_) concentrations, facilitating the exploration of their ecological consequences. Yet, nitrogen oxides (NO_x_), a class of pollutants with significant ecological and atmospheric relevance, have largely eluded field-based ecological investigations. This paper introduces the recently developed FADOE (Free-Air Diesel and Ozone Enrichment) platform, which allows the elevation of O_3_ and diesel exhaust (including NO_x_) within a field-scale context. Comprehensive information on the system's design, construction, and performance data from the 2023 summer season is presented.

• Air pollution and ecosystem functioning
• Elevated ozone and nitrogen oxides (NOx)
• Free-air exposure systems for field scale measurements.

## Graphical abstract

**Figure:**
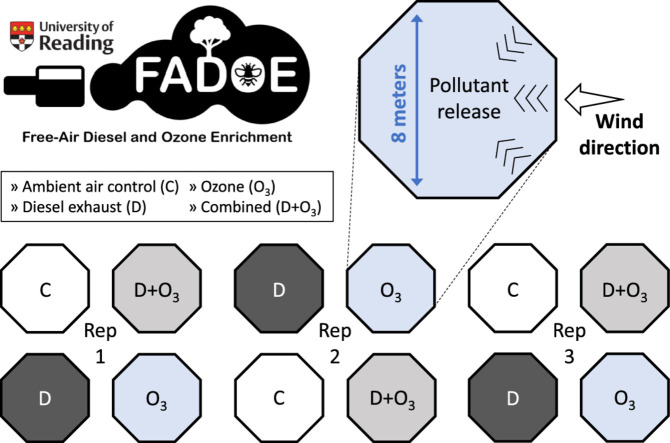

Specifications Table

**Table:** 

Subject area:	Please Select Subject Area from dropdown list
More specific subject area:	Air pollution
Name of your method:	Free-Air Diesel and Ozone Exposure (FADOE)
Name and reference of original method:	Dickson, R. E. (2000). Forest Atmosphere Carbon Transfer and Storage (FACTS-II) the Aspen Free-air CO2 and O2 Enrichment (FACE) Project: An Overview.
Resource availability:	N/A

## Background

Air pollution has become a pressing concern in the modern world, with far-reaching implications for ecosystems and their delicate processes [1]. Understanding the effects of pollutants on natural environments is crucial, but conducting field-based experiments to investigate these effects poses significant challenges. Elevating pollutant concentrations in open air conditions while maintaining a controlled and scientific approach is complex. Yet, the insights gained from such experiments, conducted under real-world conditions, are invaluable for comprehending the true impact of air pollutants on our ecosystems. In light of this, field-scale measurements have emerged as a critical avenue for advancing our knowledge of the interactions between air pollutants and the natural world. They provide a unique opportunity to observe and quantify the multifaceted consequences of pollutants on ecosystem dynamics, including critical processes such as pollination and natural pest regulation.

In atmospheric and ecological research, free-air pollutant exposure systems have been developed to successfully and consistently elevate carbon dioxide (CO_2_) and ozone (O_3_) concentrations, facilitating the examination of their ecological implications of grasslands, trees and crop plants [2], [3], [4], [5], [6], [7]). Nitrogen oxides (NO_x_) represent a pollutant class of considerable ecological and atmospheric relevance that has remained largely unexplored within field-based ecological investigations [8], [9], [10].

Understanding the intricate interactions within plant-insect communities requires careful consideration of environmental factors, particularly air pollutants like nitrogen oxides (NO_x_) and ozone (O₃).

Developing exposure systems capable of simulating realistic pollutant concentrations is crucial for disentangling the complex effects these gases have on various ecological processes. NO_x_ and O₃ exhibit distinct spatial and temporal distributions, posing unique challenges for ecological research. In urban environments, NO_x_ concentrations are typically higher due to proximity to road transport emission sources and rural areas tend to have lower levels. In the UK, average urban NO_2_ levels have reduced from 60 µg/m^3^ in the early nineties to just above 20 µg/m^3^ in the 2020s [11]. Ozone follows a different pattern, with urban areas showcasing lower concentrations (<20 ppb) compared to rural regions, with values often exceeding 30–40 ppb [12]. Additionally, both gases exhibit significant diel and seasonal fluctuations, highlighting the need for dynamic exposure systems that accurately reflect real-world conditions**.**

Historical data reveal a declining trend in NO_x_ emissions in developed nations due to stricter regulations. However, this progress is threatened by projected increases in developing countries, potentially leading to global NO_x_ emissions rising in the coming decades [13]. Predicted concentrations of O₃ are more complex, with future projections indicating potential increases in certain regions due to the interplay between precursor emissions and climate change [14]. Understanding how plant-insect communities respond to these combined changes is essential for predicting future ecosystem health and developing mitigation strategies.

Recently, we developed a Free-Air Diesel and Ozone Enrichment (FADOE) platform to allow the elevation of O_3_ and diesel exhaust (including NO_x_) in a field-scale setting. Here, we provide a comprehensive account of the design and construction of this large-scale FADOE system, along with a presentation of the performance data (Supplementary S1) derived from observations made during the summer growing season of 2023.

## Method details

### Location

The FADOE platform is located at the Sonning Farm of the University of Reading (51°28′N, 53°42′W). The platform is positioned within a managed grassland habitat, surrounded by arable fields, in an area classified as warm and temperate with daily average air temperatures ranging from 2 °C (February) to 22 °C (July), and precipitation of ca. 709 mm per year.

### Ring design and configuration

The FADOE platform consists of twelve 8 m diameter octagonal rings connected to a secure control unit container. Each ring structure is made up of 8 vertical 3 m steel scaffold tubes dug 40 cm into the ground with 3 m between each vertical scaffold tube. Each vertical tube in the ring is connected to the next by a 3 m horizontal scaffold tube at a height of 1.2 m above ground level. Eight 15-m long flexible corrugated black 25 mm internal diameter PVC aquatic hoses (TLC Electrical Supplies, UK), used for pollutant dispersal, are attached to the horizontal scaffold tubes (one flexible black hose to each horizontal scaffold tube). Along the 3 m portion of each flexible black hose that is attached to the horizontal scaffold tube, 5 mm diameter perforations are made, 20 cm apart, to allow pollutant release. The end of each flexible hose attached to a scaffold tube is sealed with a black plastic cap. The other end of each hose is connected to an eight-port release manifold (see pollutant delivery system section below), one manifold per ring.

**Fig. 1:**
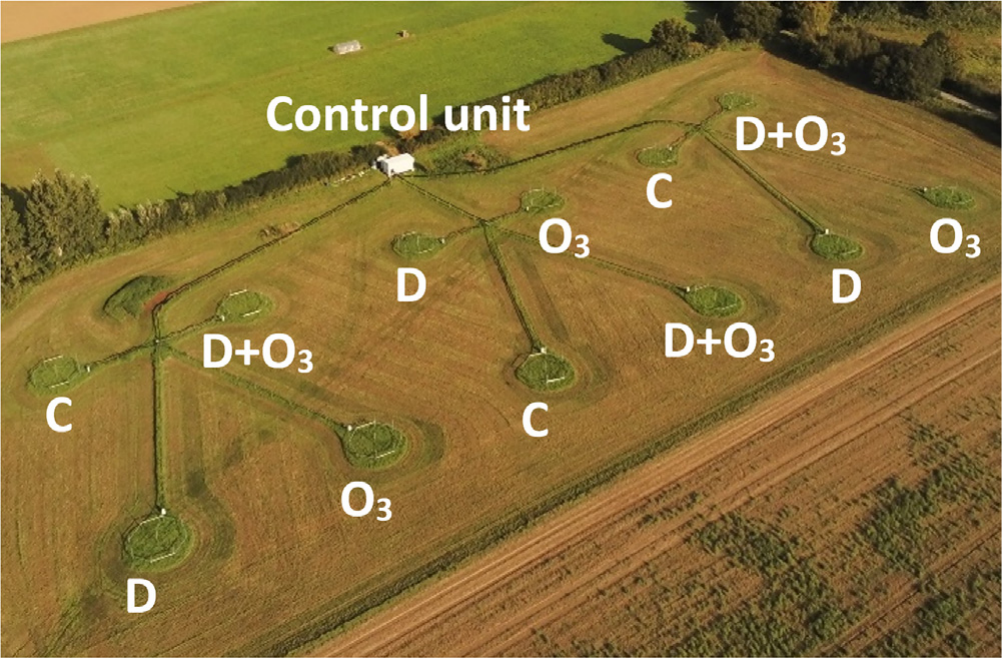
Free-Air Diesel and Ozone Enrichment (FADOE) platform, the first dedicated field-based facility built to facilitate investigations into the ecological impacts of combinations of air pollutants. The twelve 8 m diameter rings emit realistic quantities of diesel exhaust and ozone pollutants from perforated tubes surrounding the rings. Three of the rings are assigned to each of four treatments – ambient control (C), ozone (O_3_), diesel exhaust (D) and diesel exhaust and ozone combined (*D*+O_3_).

Three rings were assigned to each of four treatments – ambient control (C), ozone (O_3_), diesel exhaust (D) and diesel exhaust and ozone (*D*+O_3_). The rings are separated such that the center of each ring is positioned at least 30 m from the centre of other rings to prevent cross contamination of pollutants between rings. Rings are grouped into four numerical units (1–4, 5–8, 9–12) with each unit having all treatment regimens (Fig. 1).

### Pollutant delivery system

The pollutant delivery system is housed in a secure central control unit, consisting of a modified 20 ft steel shipping container (this includes a bespoke interior designed to house the control systems detailed below; Supplementary S2 and S3). Twelve R3105 series (Gast, USA) treatment delivery pumps are used to deliver ambient air/diesel exhaust to the FADOE rings via 50 mm (internal diameter) HDPE conduit. Diesel exhaust is piped from a diesel generator (SSDK12W Kubota 12.6 kVA, SGS Engineering Ltd, UK) and passed through a metal conduit for heat dissipation and then a conditioning volume (consisting of three connected 44-gallon steel drums each with a tap to facilitate draining) to remove excess water vapor. Excess diesel exhaust is ducted away from the site to ensure that there is no impact on the measurements made in each treatment ring.

At each ring an eight-port release manifold (see ring design section above) with solenoid valves (Asco series L133, OEM Automatic, UK) is used to disperse the treatment air from the upwind sides of the ring, through 25 mm (internal diameter) PVC hose, to ensure consistent flow of treatment gases across the rings. Outlets from the release manifold valves are connected to the 25 mm PVC hoses that surround the rings. In control (C) rings, the treatment air supplied is ambient air only, which is taken in at the central control unit. For D and *D*+O_3_ rings ambient air and diesel exhaust are blended separately for each ring at the control unit using three-way mixing valves (VRG131 valves and ARA600 proportional actuators, ESBE, Sweden) to achieve the target NO_x_ concentration. Images of the individual rings and control systems are shown in Supplementary S4.

Three O_3_ generators (CD2000P, ClearWater Tech, USA) (one for each group of four rings) are used to deliver elevated O_3_ to the control manifolds of O_3_ and *D*+O_3_ rings via 3/8″ PTFE tubing. The O_3_ generators are fed with ambient air from a diaphragm compressor (87R637–101-N470X, Gast, USA). The outlet of each O_3_ generator is divided between two delivery lines, each one supplying a pair of O_3_ and *D*+O_3_ treated rings, and each line's flow is regulated with a rotameter to ensure even delivery of O_3_ to each ring.

For O_3_ and *D*+O_3_ treatment rings the PTFE O_3_ delivery tubing is connected to the 50 mm delivery conduit immediately upstream of the release manifold with a perforated tube inserted into the conduit to ensure the O_3_ supply is well mixed with the treatment air.

WSD-V wind speed & direction sensors (Measurement Systems Ltd.) are used in the center of each ring to control the direction of treatment release so that treatment air is always released from the three upwind sides of each ring. Where windspeeds are below the instrument's measurement threshold (0.2 m s^−1^) then alternate sides of the ring are opened on a five second cycle time to disperse treatment air around the ring.

### Monitoring and data acquisition

The FADOE system is controlled by a LabVIEW (National Instruments, TX USA) based control program. This control program starts and stops all of the system's equipment on a predetermined cycle that can be adjusted to run at different times on each day of the week to account for typical cycles in pollutants that may be expected due to human activity such as busy commuting times. Analogue voltage signals from the wind sensors are recorded on USB-201 data acquisition devices (Measurement Computing Corporation, MA USA) and read by the control program.

One Teledyne-API T400 UV absorption O_3_ analyzer and one Teledyne-API T200P photolytic NO_2_/NO/NO_x_ analyzer (ET enviro, UK) are used to measure concentrations of pollutants from sampling points located at the center of each ring. The analyzers are housed in the central control unit and filtered air samples (filtered using 47 mm PTFE 1.2 μm membranes, Cole-Parmer, UK) are delivered to the analyzers via 1/4” PTFE sample lines and a 12-port stainless steel valve manifold for sample selection. Sample lines from each ring are continually purged between measurements to minimize the time between measurements from different rings. Data from the wind sensors and gas analysers are recorded and processed via the LabVIEW control program. Results are used to determine the feedback parameters in adjusting diesel and O_3_ treatment release and to control the ring side manifold valves. Ring side manifold valves are switched via a networked relay control unit located at each ring side manifold (UDP eight channel relay controller, KMTronic, Bulgaria).

The concentration of O_3_ delivered to each ring group is adjusted by a 4–20 mA control signal provided to each O_3_ generator by a USB-3104 analogue current output device (Measurement Systems Ltd, UK).

Between 5 May and 7 July 2023, analyser purge time and sampling time were set at 120 s and 60 s per ring, respectively. During each sampling period, pollutant concentrations taken every second are averaged and recorded automatically in csv format (Supplementary S1). Average NO_x_, NO, NO_2_ and O_3_ concentrations (ppb ± standard deviation), wind speed and wind direction are recorded for each ring sequentially.

### Representative output

**Fig. 2:**
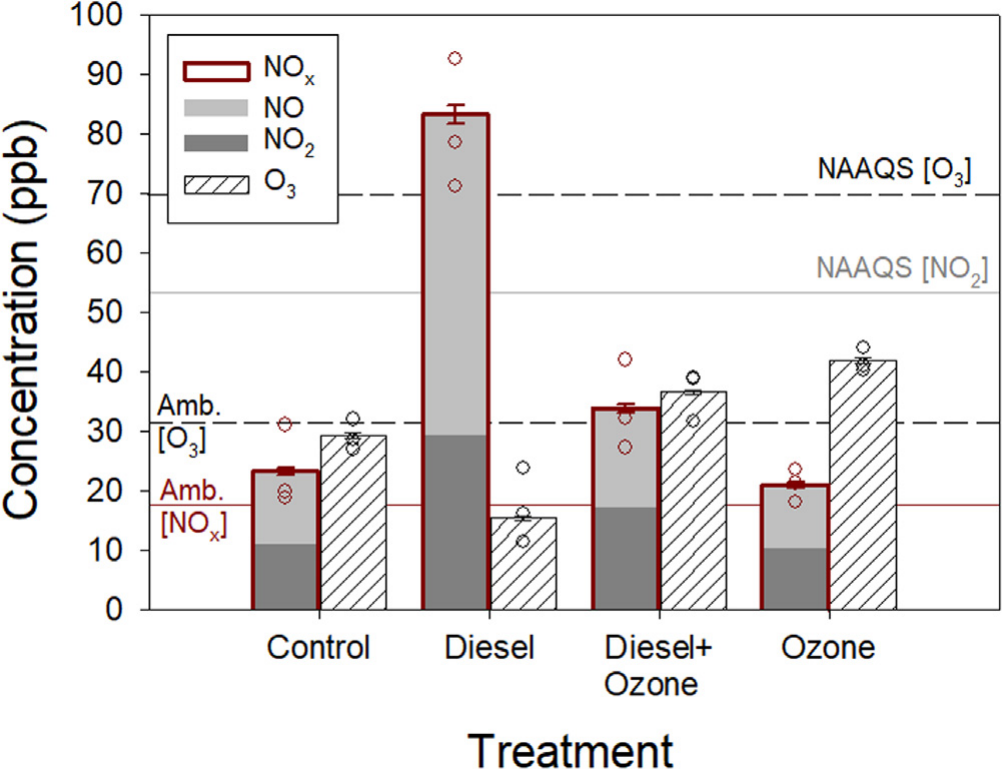
Mean concentrations (±SE) of nitrogen oxides (NO_x_ = NO + NO_2_) and ozone (O_3_) within treatments. Red bars (NO_x_ concentrations) include stacked concentrations of nitric oxide (NO) and nitrogen dioxide (NO_2_). Circles are average concentrations from individual FADOE rings (*N* = 3 per treatment). Target concentrations of NO_x_ and O_3_ were set at 90 ppb and 70 ppb, respectively. Ambient background concentrations (Amb.) from the nearest monitoring station in Reading, UK (London Road and New Town for NO_x_ and O_3_, respectively) and National Ambient Air Quality Standards (NAAQS) set by the United States Environmental Protection Agency are visualised in ppb as horizontal lines.

During the summer of 2023, our investigations within the FADOE platform enabled the deliberate elevation of the mean NO_x_ concentration to an average level of 83 ppb, which corresponded to more than fourfold of the ambient levels. Additionally, the average O_3_ concentrations in the ozone plots were elevated to a magnitude 1.5 times greater than ambient levels. Notably, the Diesel + O_3_ plots exhibited significantly reduced concentrations of both NO_x_ and O_3_, attributed to chemical reactions between these pollutants, as illustrated in Fig. 2. Previous research [8] utilizing an earlier FADOE temporary prototype revealed that moderate elevations in NO_x_ and O_3_ levels led to a striking 90 % reduction in flower visitation by pollinators, signifying an unexpectedly severe adverse impact on insect-mediated pollination. The prototype facility also demonstrated deleterious effects air pollution on natural enemies of herbivores and ground-dwelling insects [[9], [10]]. The current FADOE system, with increased replication and by dynamically controlling for changes in wind speed/direction, enables the advancement of knowledge into the real-world impacts of diesel exhaust and O_3_, individually and interactively, on biological and ecological processes and/or atmospheric chemistry.

## Ethics statement

Not applicable

## CRediT authorship contribution statement

**Adedayo O. Mofikoya:** Writing – original draft, Data curation. **Laura James:** Data curation, Writing – review & editing. **Neil J. Mullinger:** Conceptualization, Funding acquisition, Software, Validation, Writing – review & editing. **James M.W. Ryalls:** Conceptualization, Funding acquisition, Visualization, Data curation, Writing – review & editing. **Robbie D. Girling:** Conceptualization, Funding acquisition, Data curation, Writing – review & editing, Supervision.

## Declaration of competing interest

The authors declare that they have no known competing financial interests or personal relationships that could have appeared to influence the work reported in this paper.

## Data Availability

Data will be made available on request.

## Appendix Supplementary materials

Image, application 1

Image, application 2

Image, application 3

Image, application 4
