# Supplementary material for: A novel free-air diesel and ozone enrichment (FADOE) research platform
Source: MethodsX. 2024 Feb 27;12:102635. doi: 10.1016/j.mex.2024.102635 (PMC10918276; doi:10.1016/j.mex.2024.102635)
Supplement: Supplementary file 2 [file mmc2.pdf]

DO NOT SCALE - IF IN DOUBT ASK
ALL DIMENSIONS IN MM UNLESS STATED OTHERWISE THIS DRAWING IS THE PROPERTY OF S. JONES CONTAINERS LTD
AND SHOULD NOT BE COPIED OR FORWARDED TO A THIRD PARTY WITHOUT CONSENT

3rd ANGLE PROJECTION

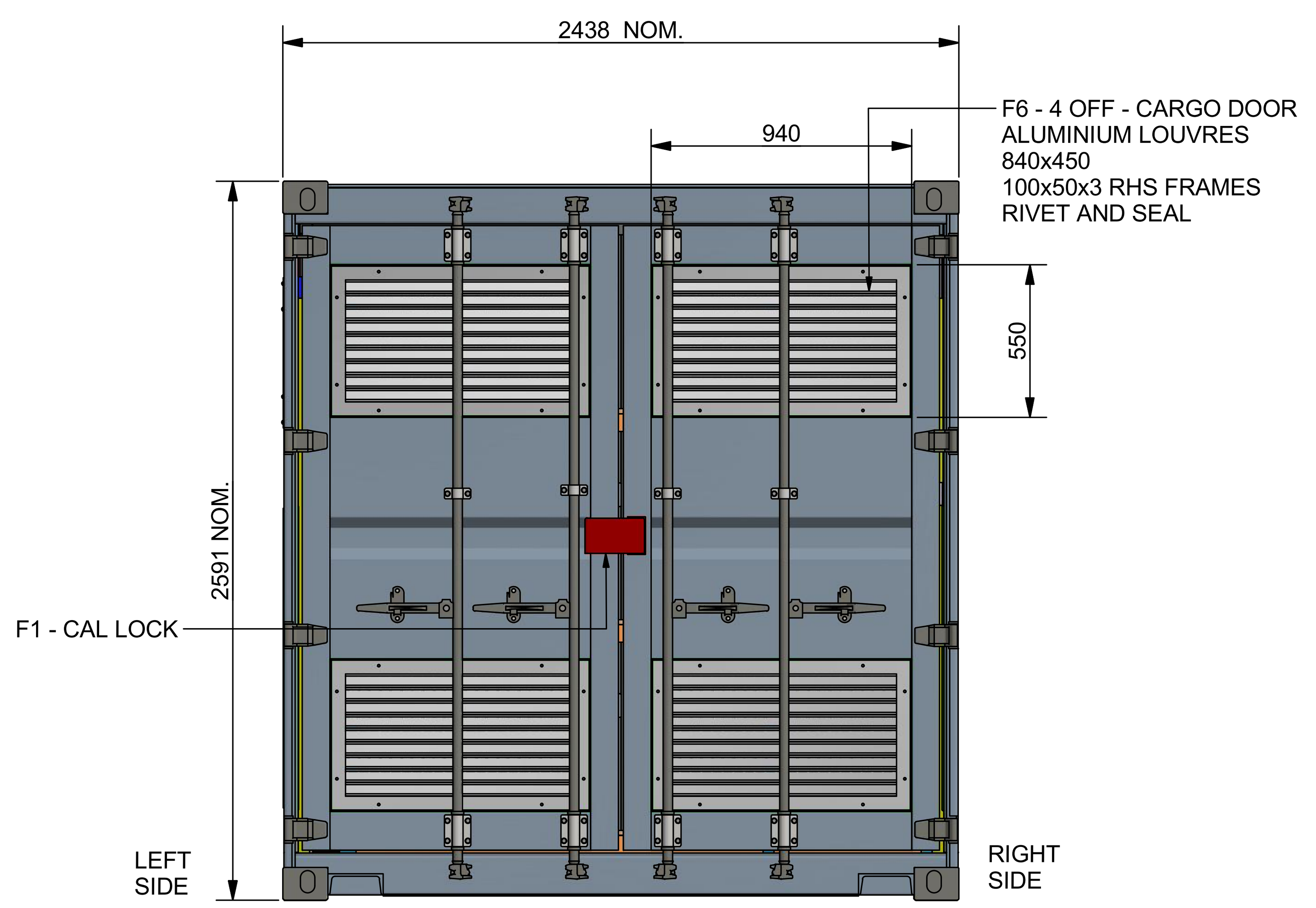

REAR VIEW (1 : 20)

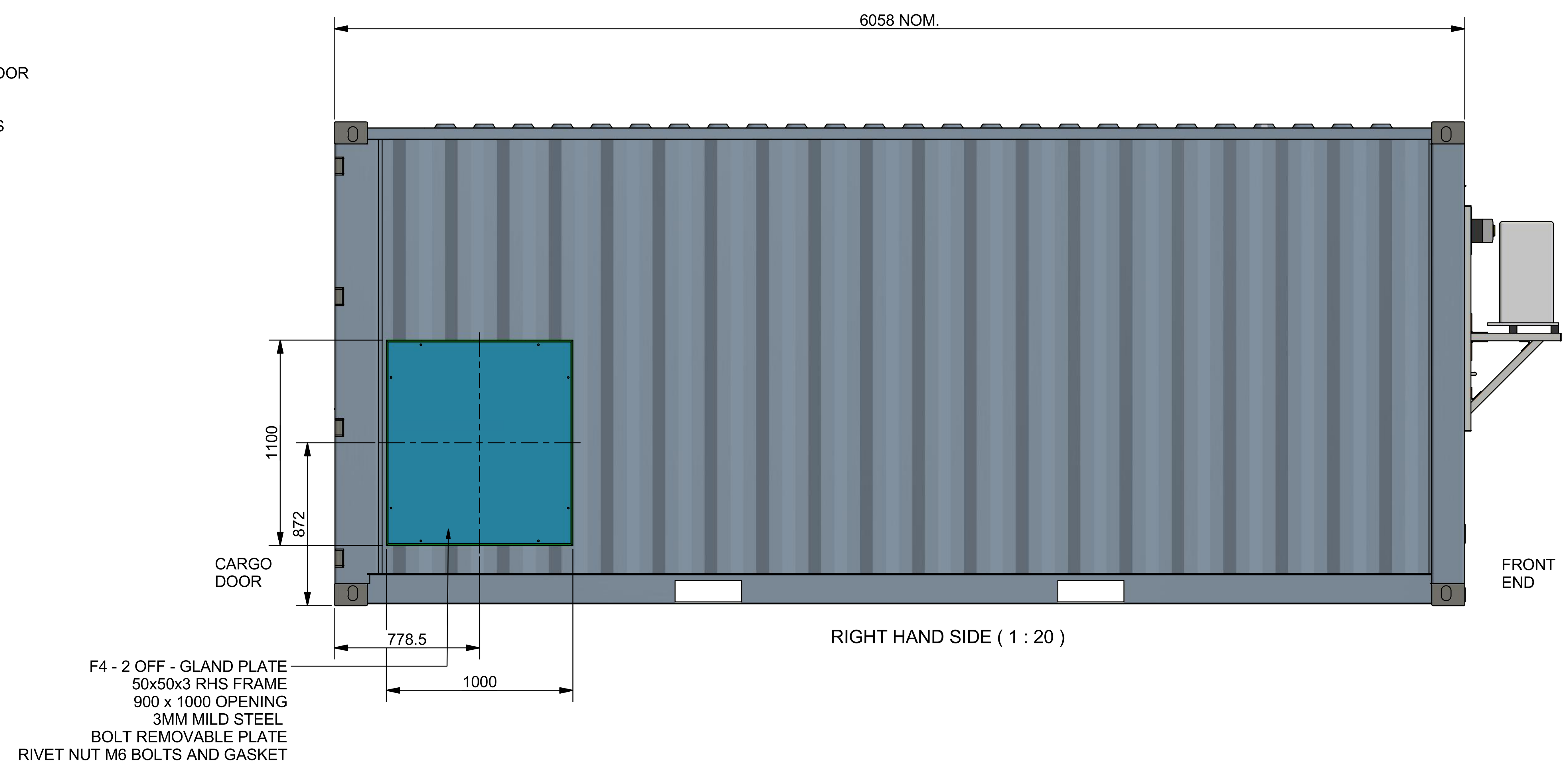

RIGHT HAND SIDE (1 : 20)

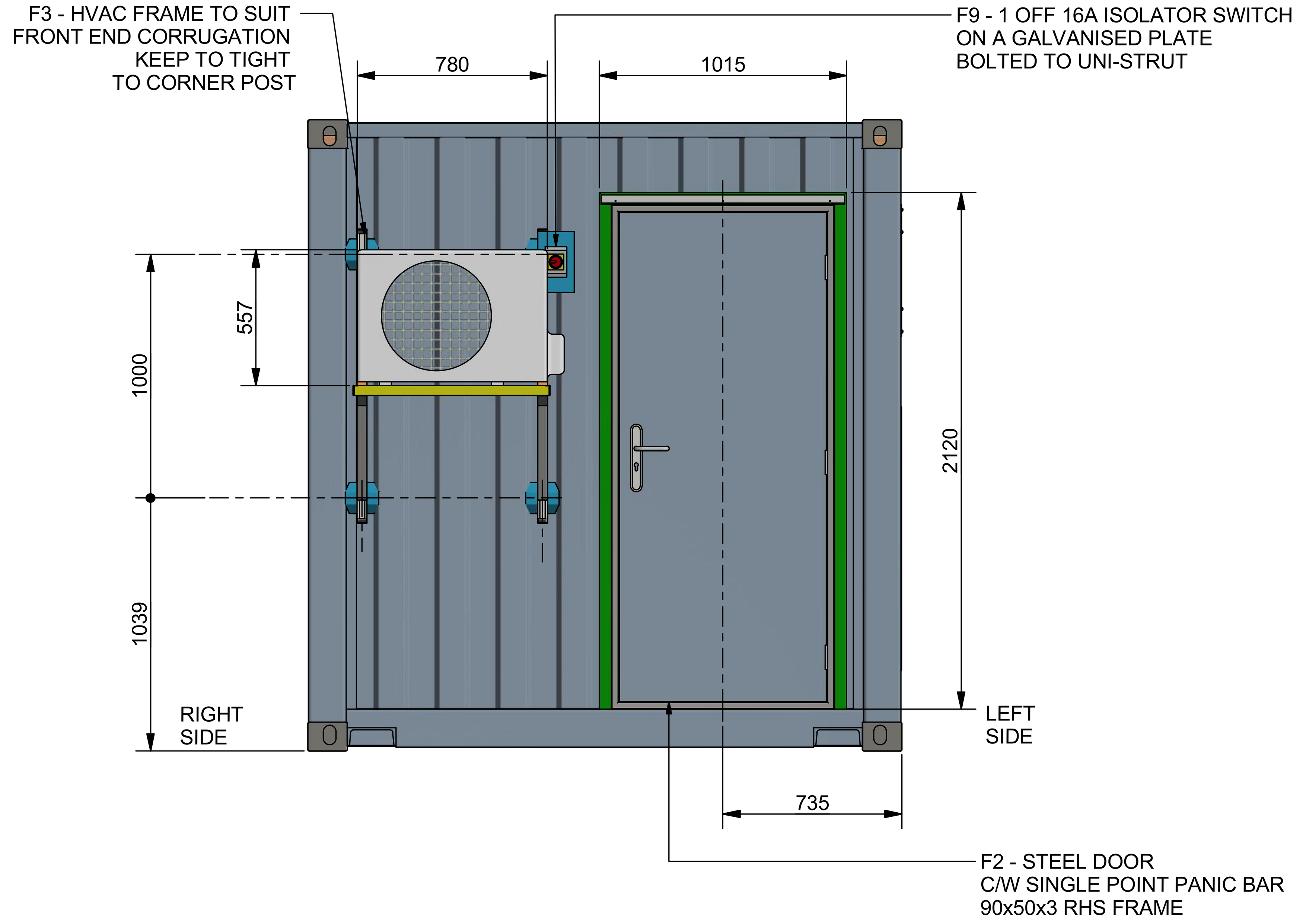

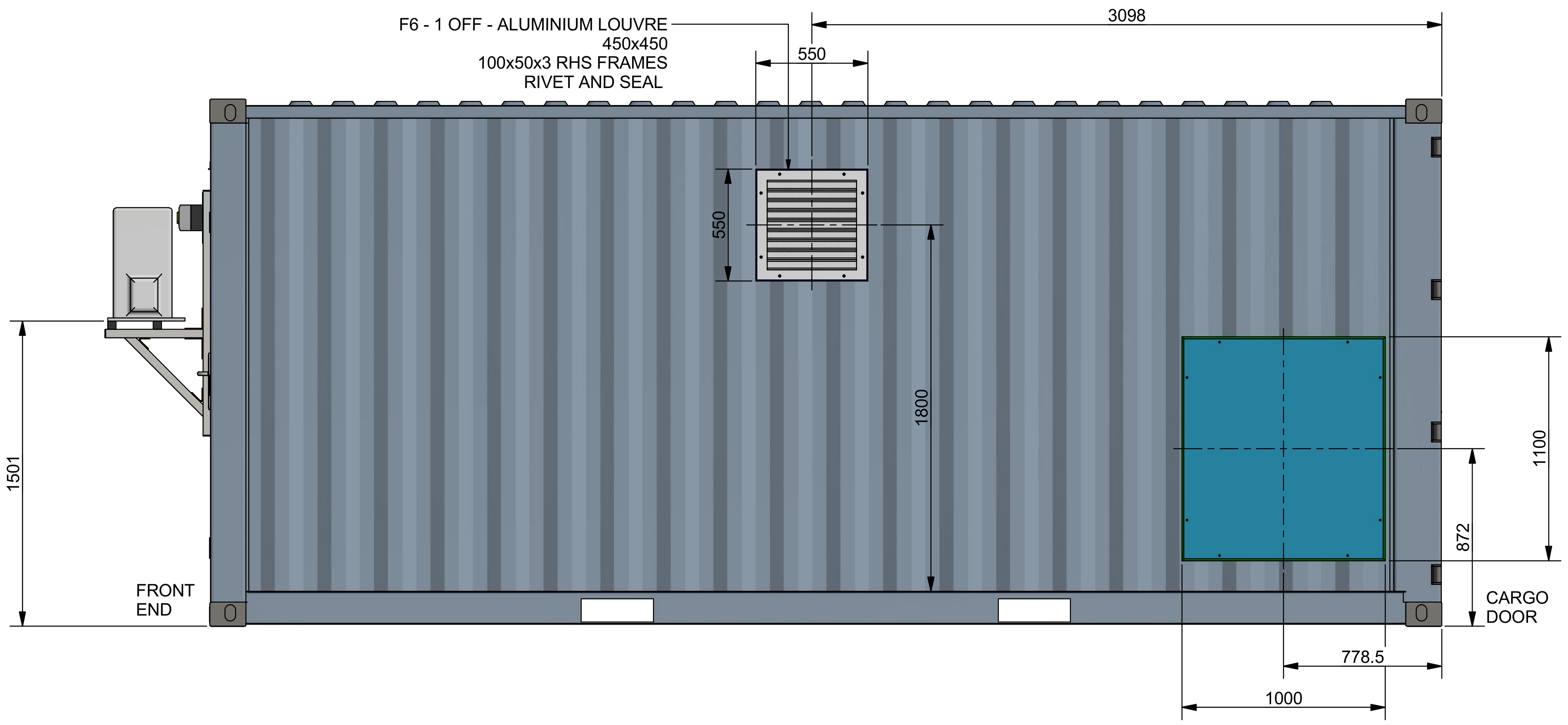

NOTES

* - DIMENSIONS THAT CAN VARY DUE TO CONTAINER MANUFACTURE

RAL - 9003.

REV	DATE	AMENDMENT
P1	14/01/2020	FOR APPROVAL

CONTAINER NUMBER

CLIENT/PROJECT
CEH UNIVERSITY OF READING

SJONES CONTAINERS
ANGLIAN ROAD
ALDRIDGE
WALSALL
WEST MIDLANDS WS9 8ET

TEL :- 01922 741751
FAX :- 01922 741753

TITLE
INTERNAL GA DETAILS

DRAWN	DATE
DJP	06/01/2020

QUOTE REF	SHEET

DRAWING No.	ISSUE
81190-01	P1

DO NOT SCALE - IF IN DOUBT ASK
ALL DIMENSIONS IN MM UNLESS STATED OTHERWISE THIS DRAWING IS THE PROPERTY OF S. JONES CONTAINERS LTD
AND SHOULD NOT BE COPIED OR FORWARDED TO A THIRD PARTY WITHOUT CONSENT

3rd ANGLE PROJECTION

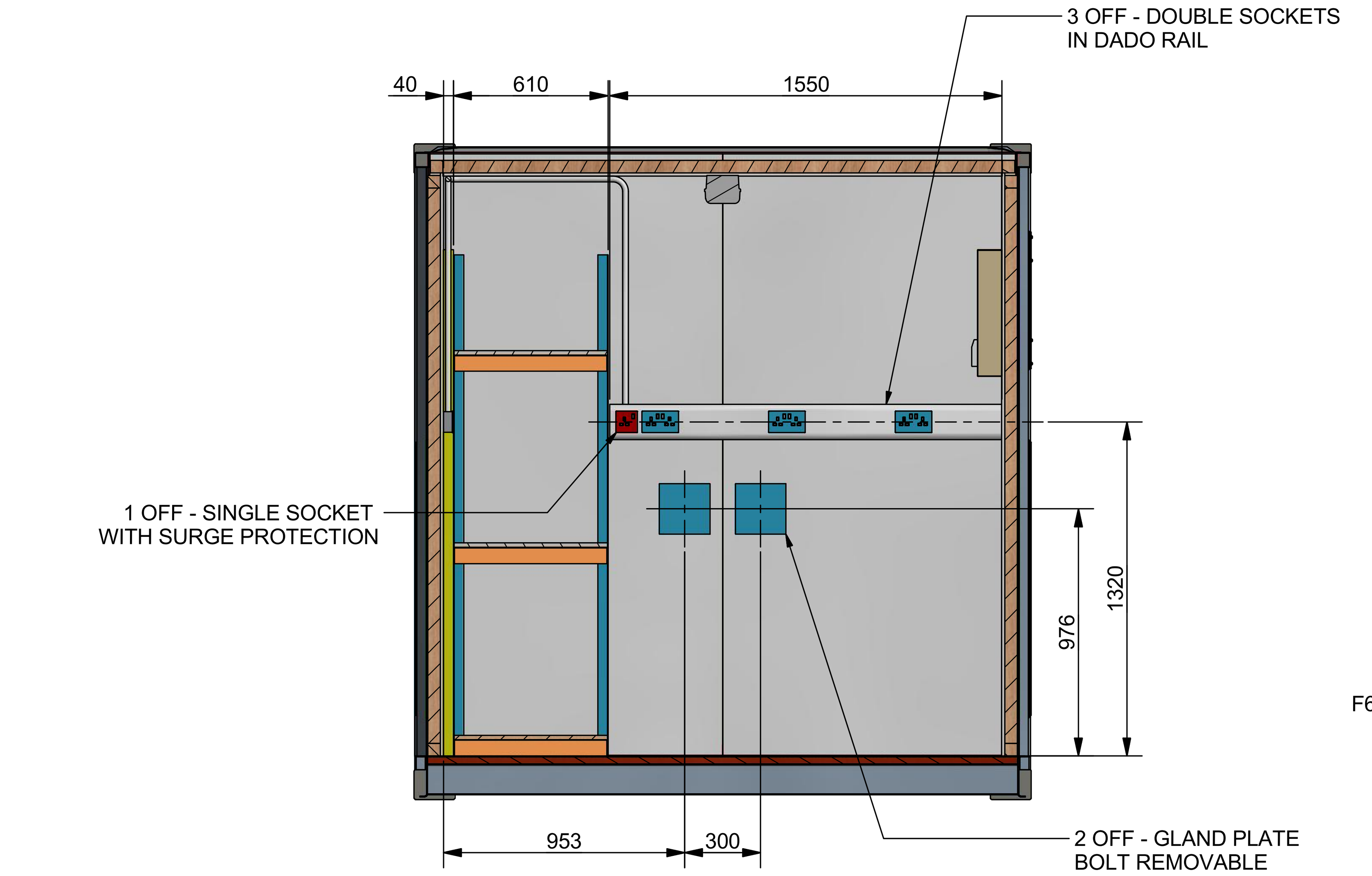

SECTION H-H
(1 : 20)

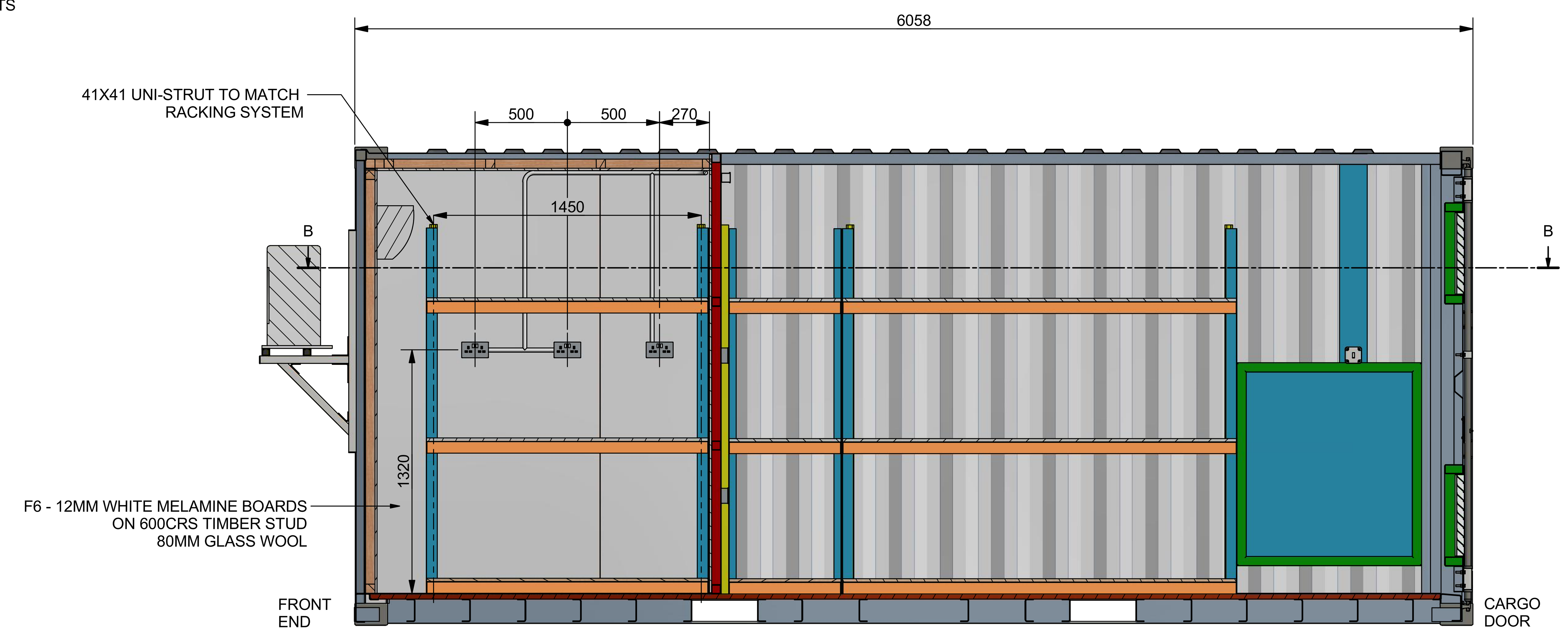

SECTION A-A
(1 : 20)

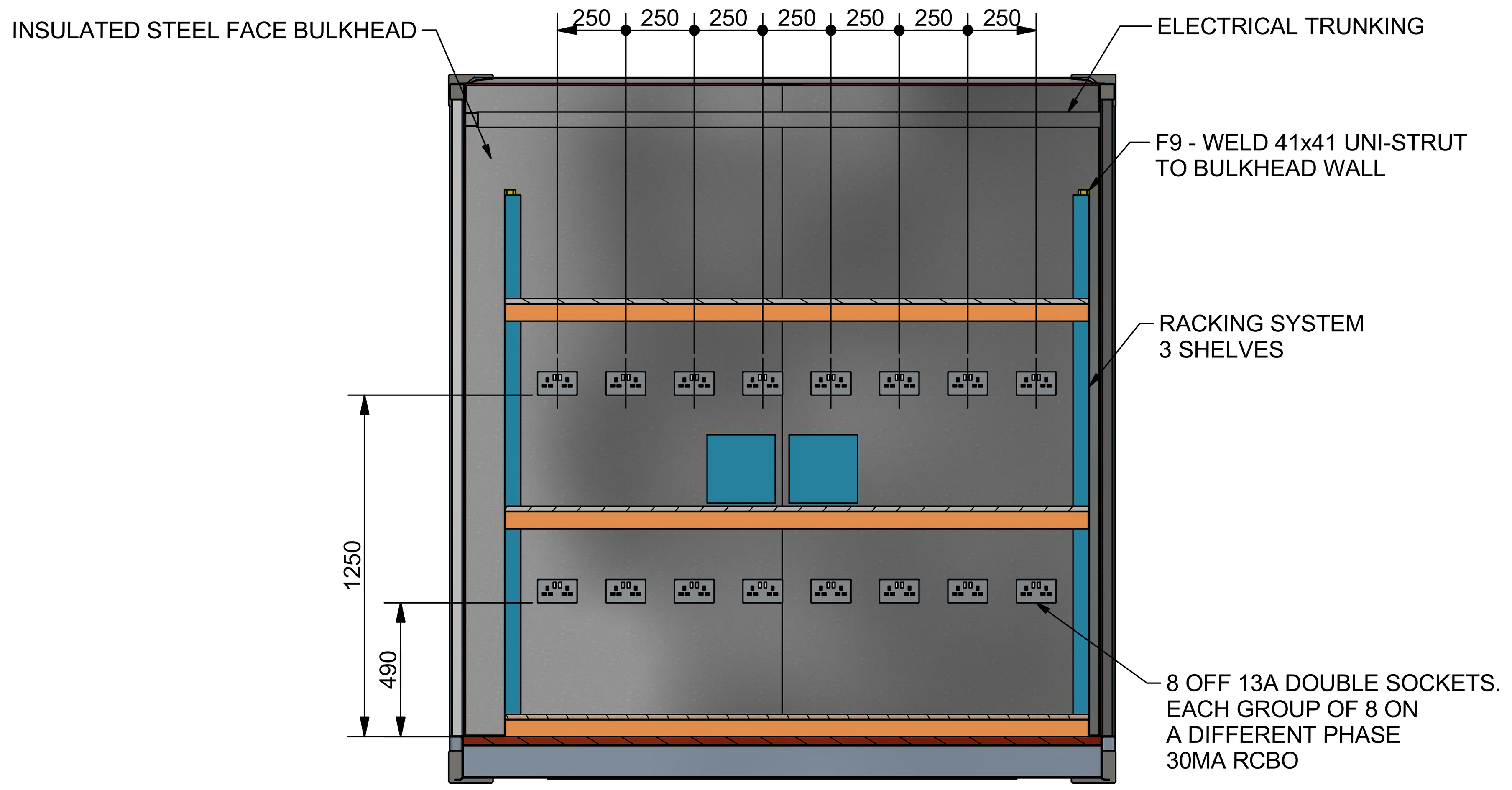

SECTION J-J
(1 : 20)

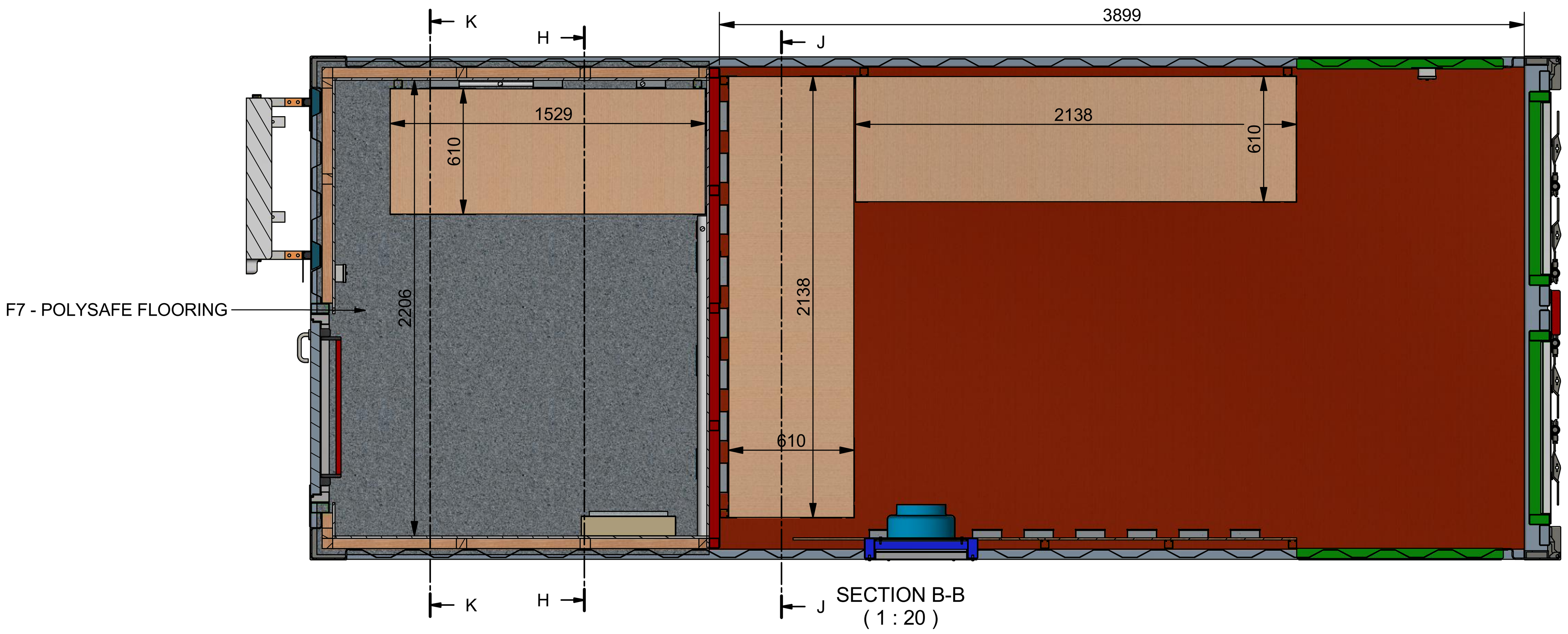

SECTION B-B
(1 : 20)

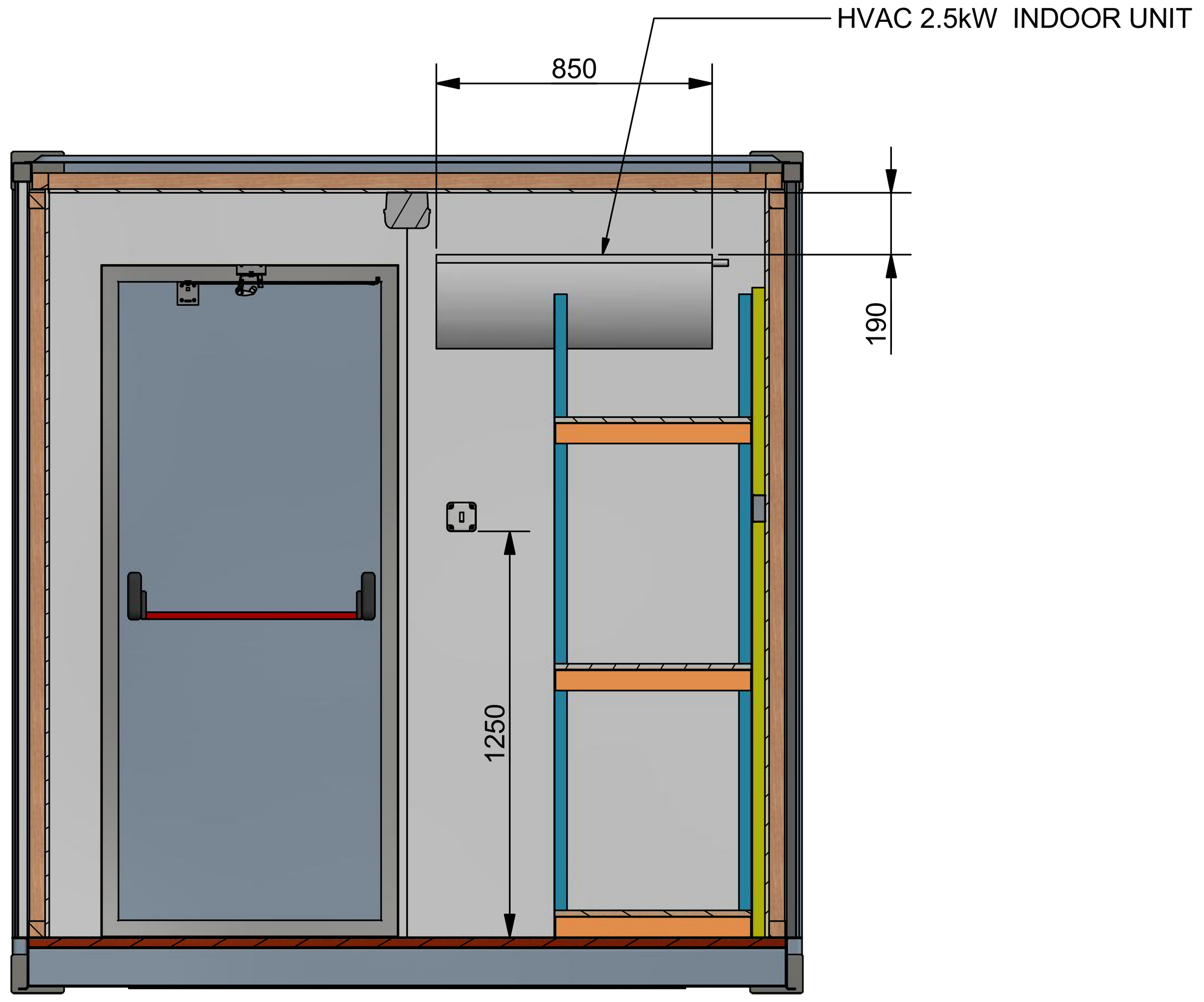

SECTION K-K
(1 : 20)

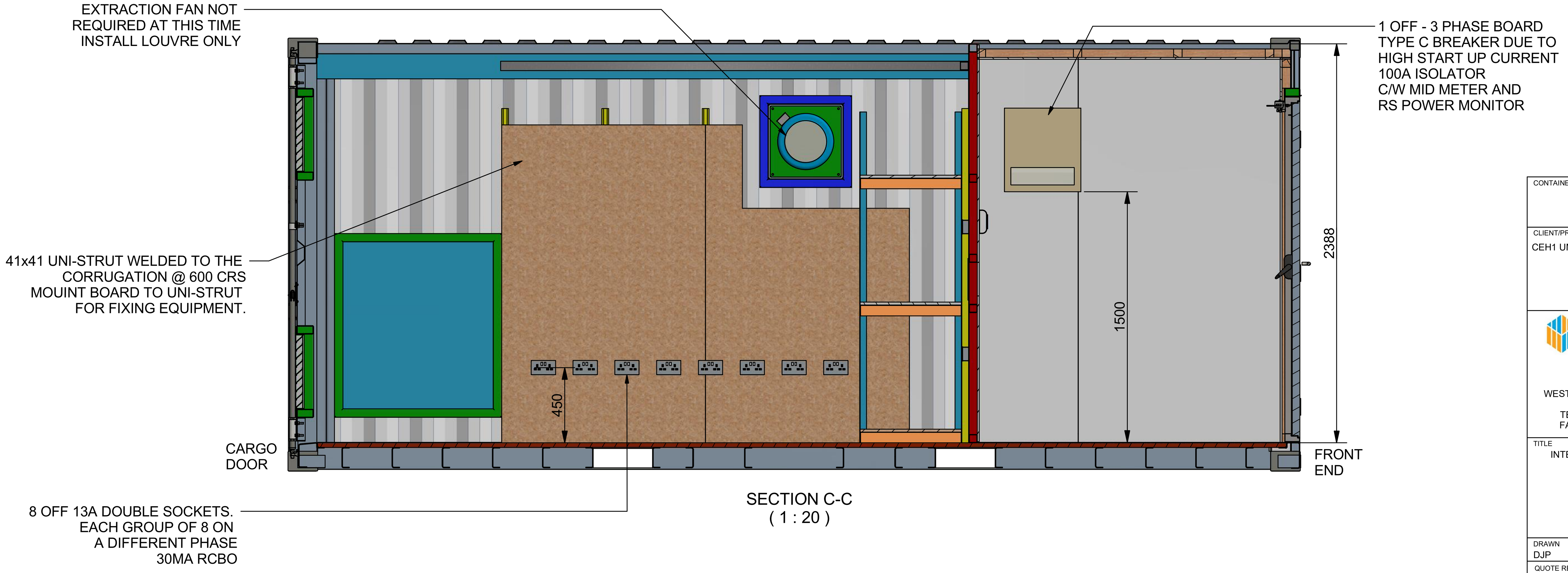

SECTION C-C
(1 : 20)

NOTES

* - DIMENSIONS THAT CAN VARY DUE TO CONTAINER MANUFACTURE

REV	DATE	AMENDMENT
P1	14/01/2020	FOR APPROVAL

CONTAINER NUMBER

CLIENT/PROJECT
CEH1 UNIVERSITY OF READING

SJONES CONTAINERS
ANGLIAN ROAD
ALDRIDGE
WALSALL
WEST MIDLANDS WS9 8ET

TEL :- 01922 741751
FAX :- 01922 741753

TITLE
INTERNAL GA DETAILS

DRAWN	DATE
DJP	06/01/2020
QUOTE REF	SHEET
DRAWING No. 81190-02	ISSUE P1
