# Supplementary material for: A novel free-air diesel and ozone enrichment (FADOE) research platform
Source: MethodsX. 2024 Feb 27;12:102635. doi: 10.1016/j.mex.2024.102635 (PMC10918276; doi:10.1016/j.mex.2024.102635)

Free-Air Diesel and Ozone Enrichment (FADOE) ring air delivery system

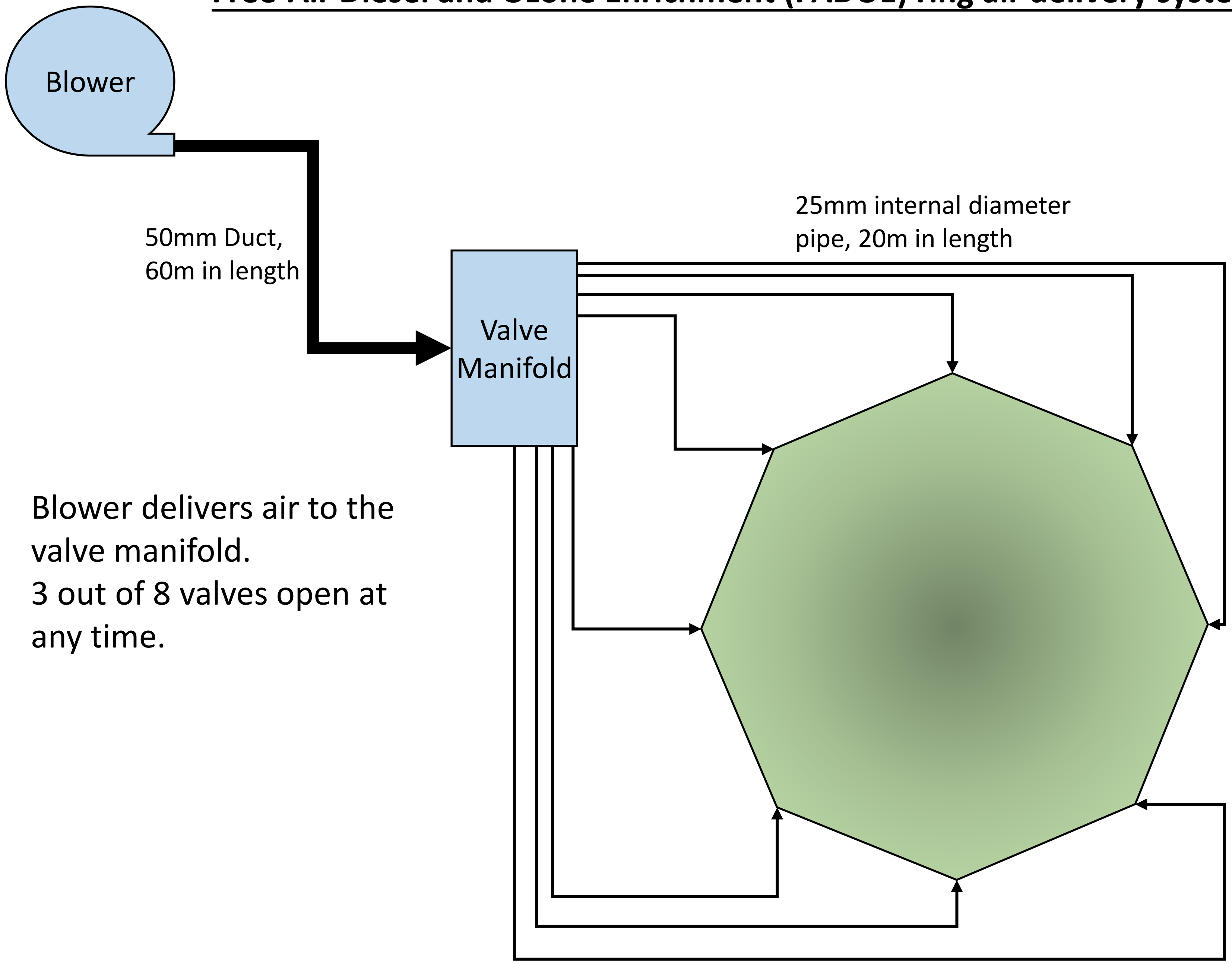

- Blower delivers air to the valve manifold.
- 3 out of 8 valves open at any time.

Control/O_3 FADOE system

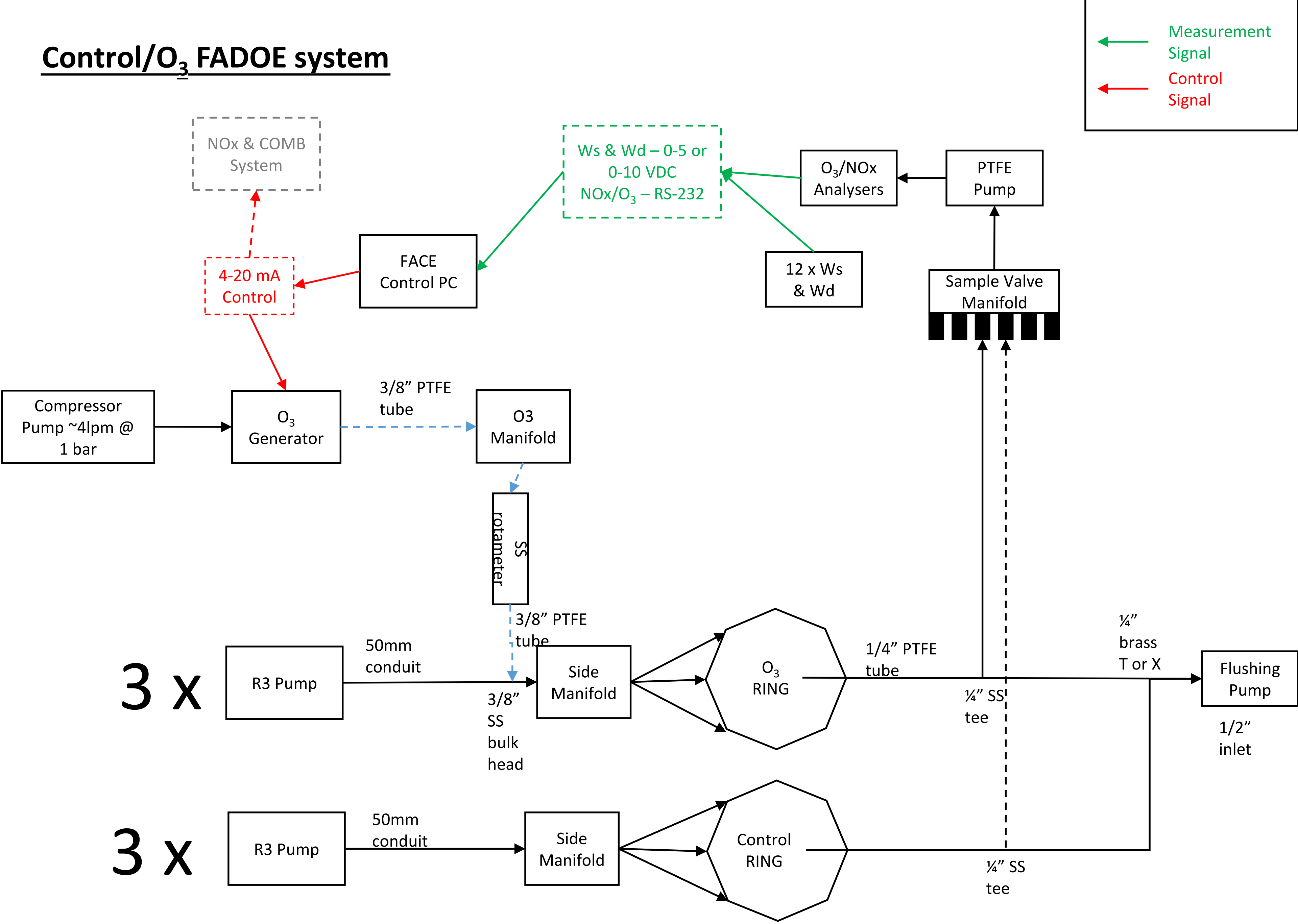

NOx/Combined FADOE System

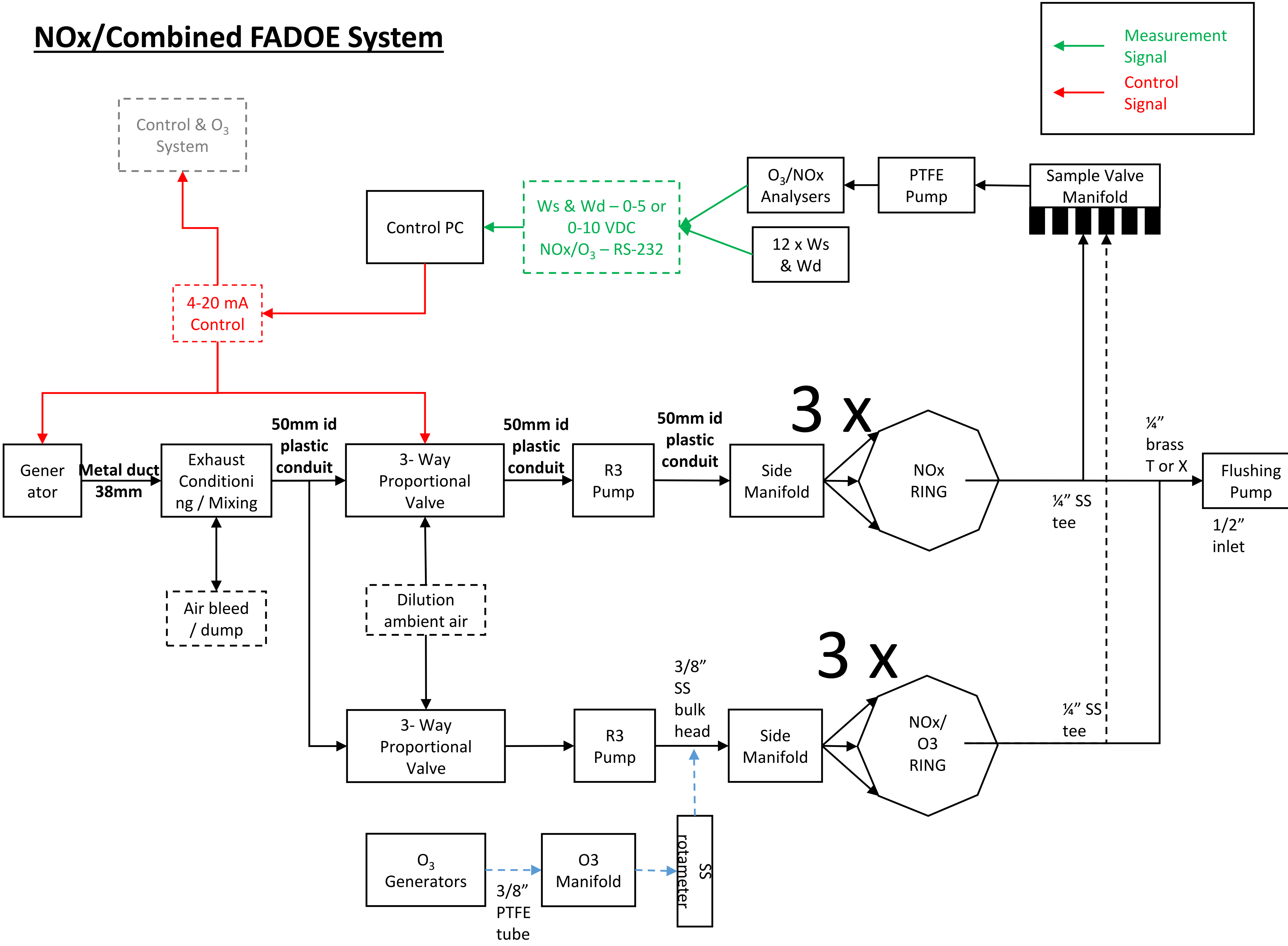

Ozone Distribution System

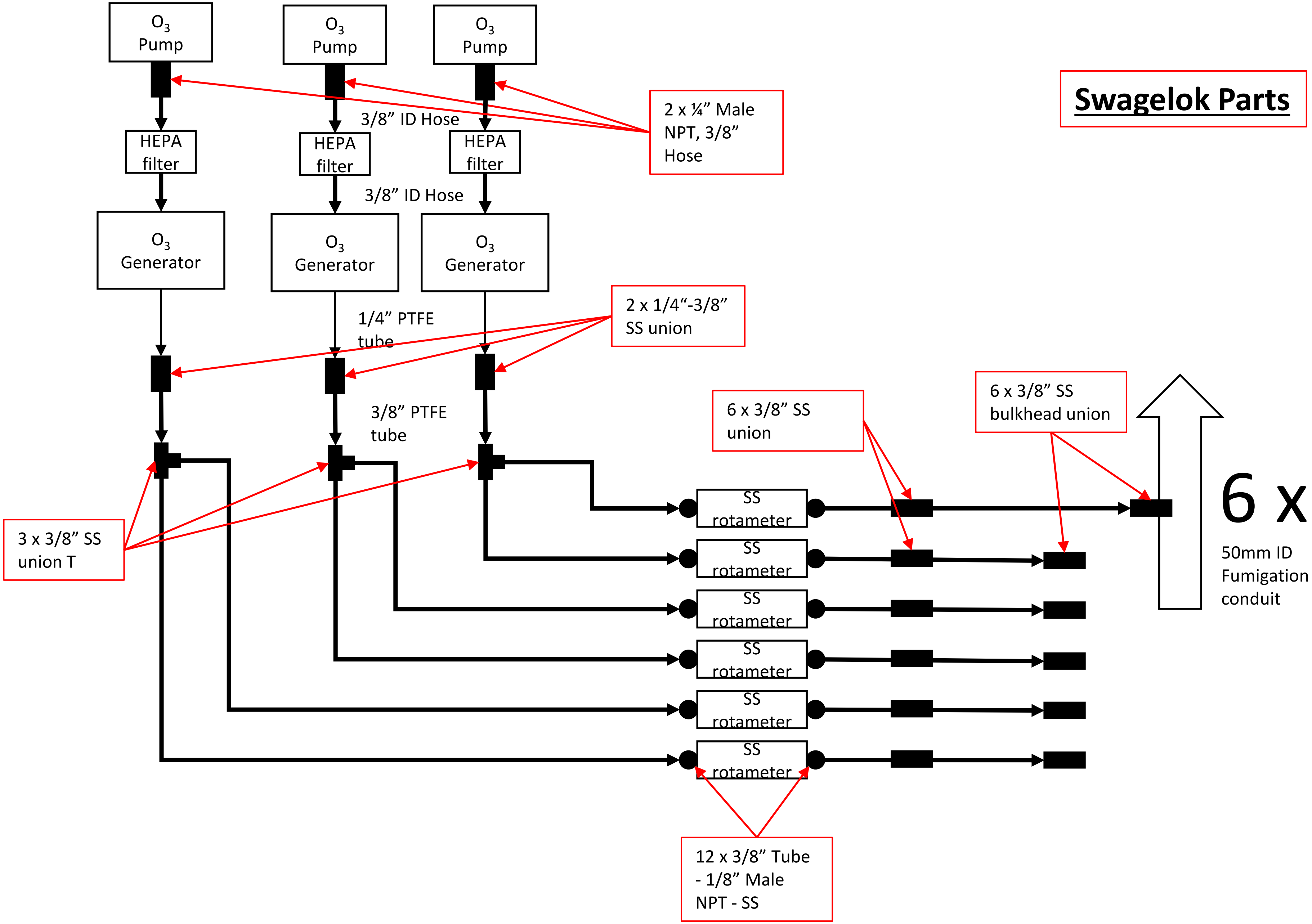

Sampling Schematic (for sampling gas concentrations from the centre of each ring)

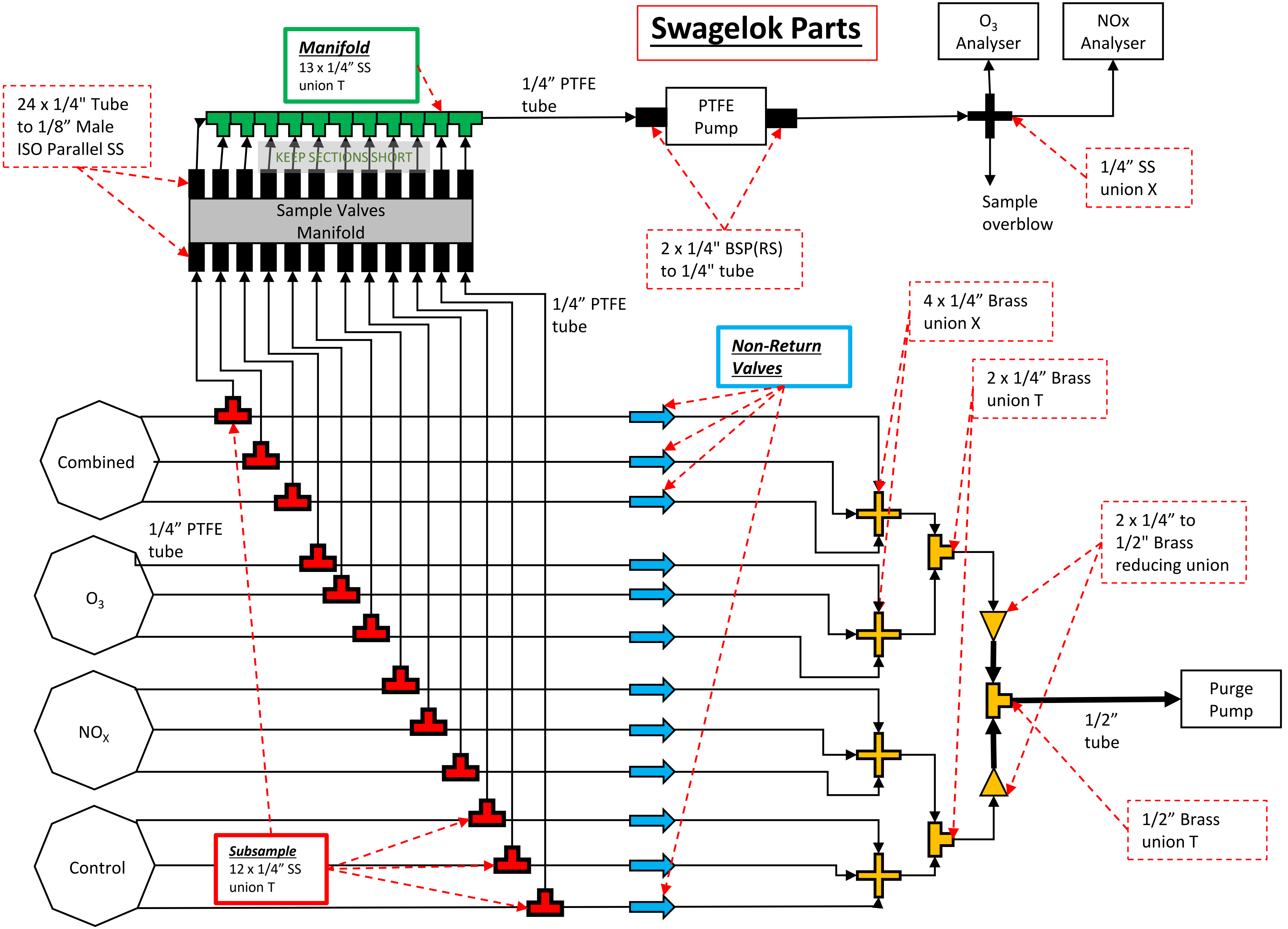

<u>**Sampling Manifold Schematic**</u> (for sampling gas concentrations from the centre of each ring)

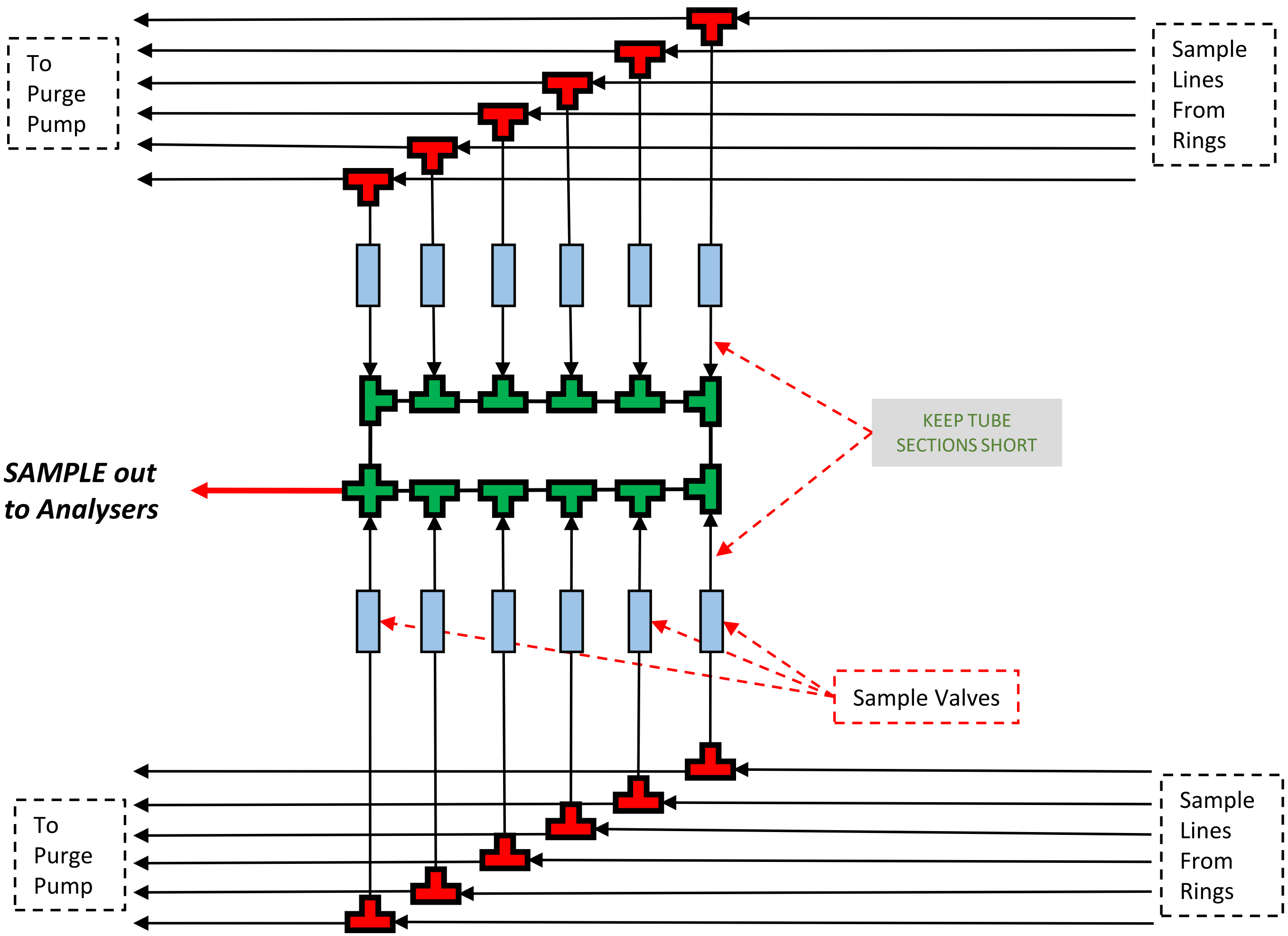

Logging equipment

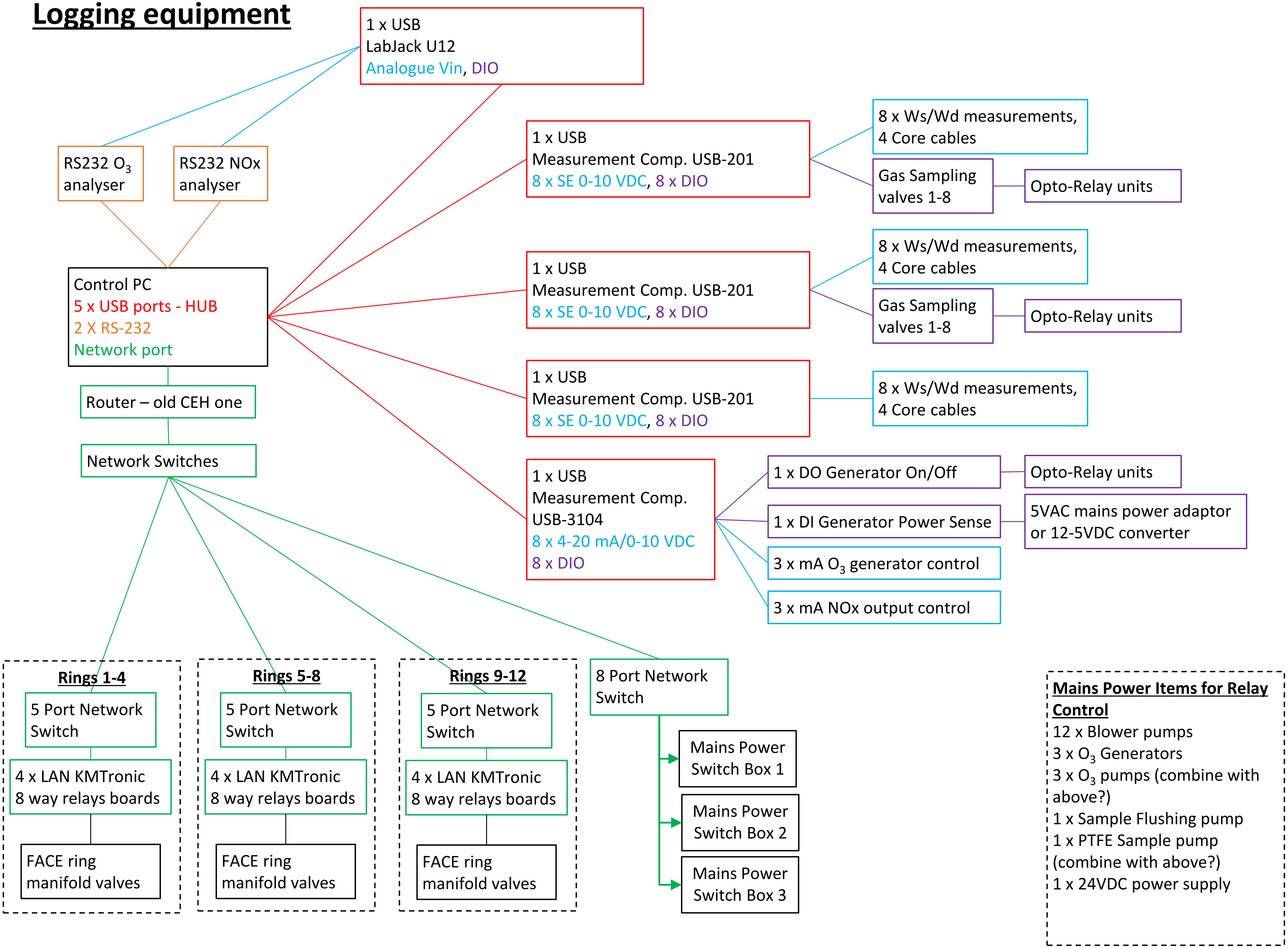

Power Schematic

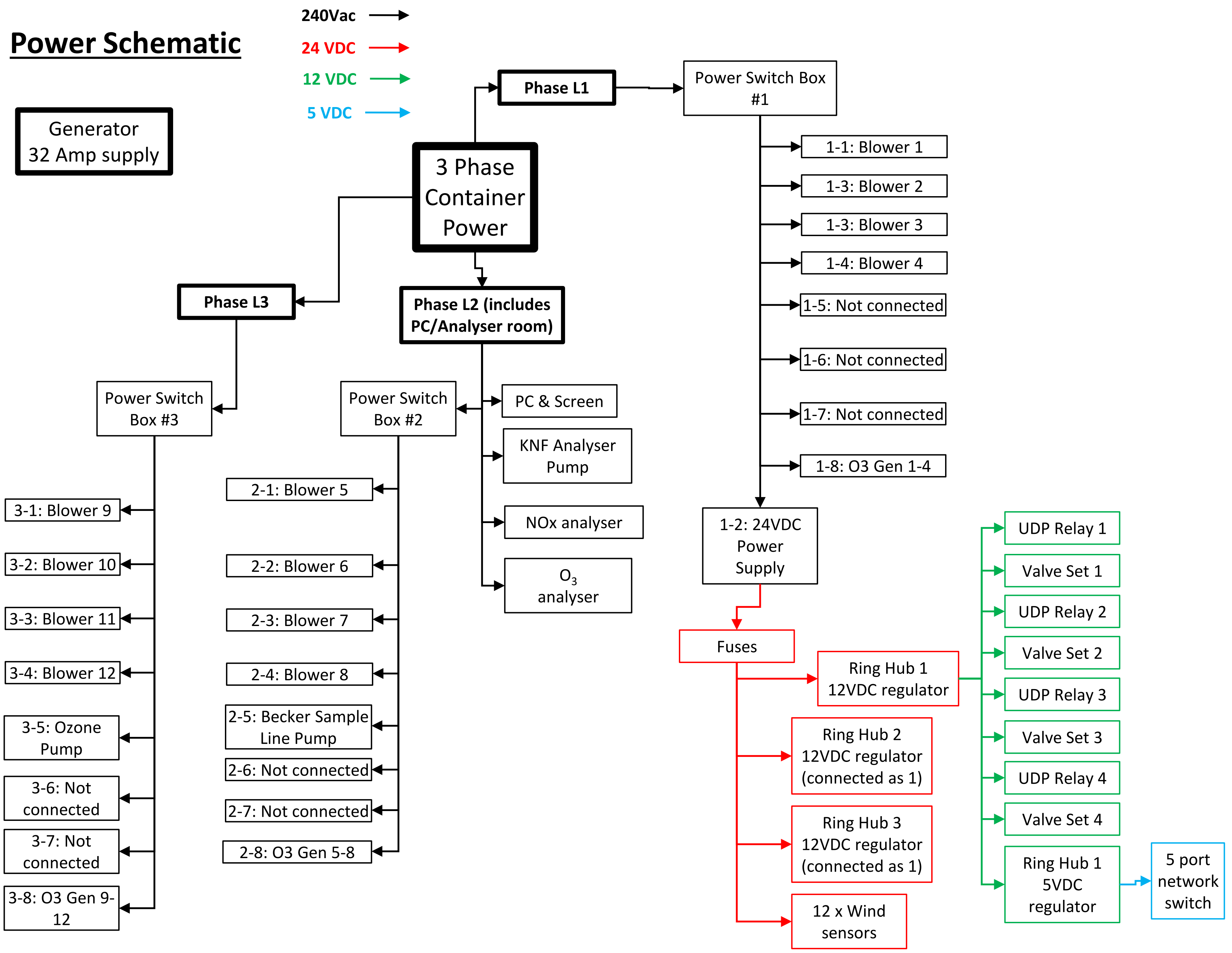

8-Way Contactor Unit – 12VDC connections to control power to equipment (e.g. pumps)

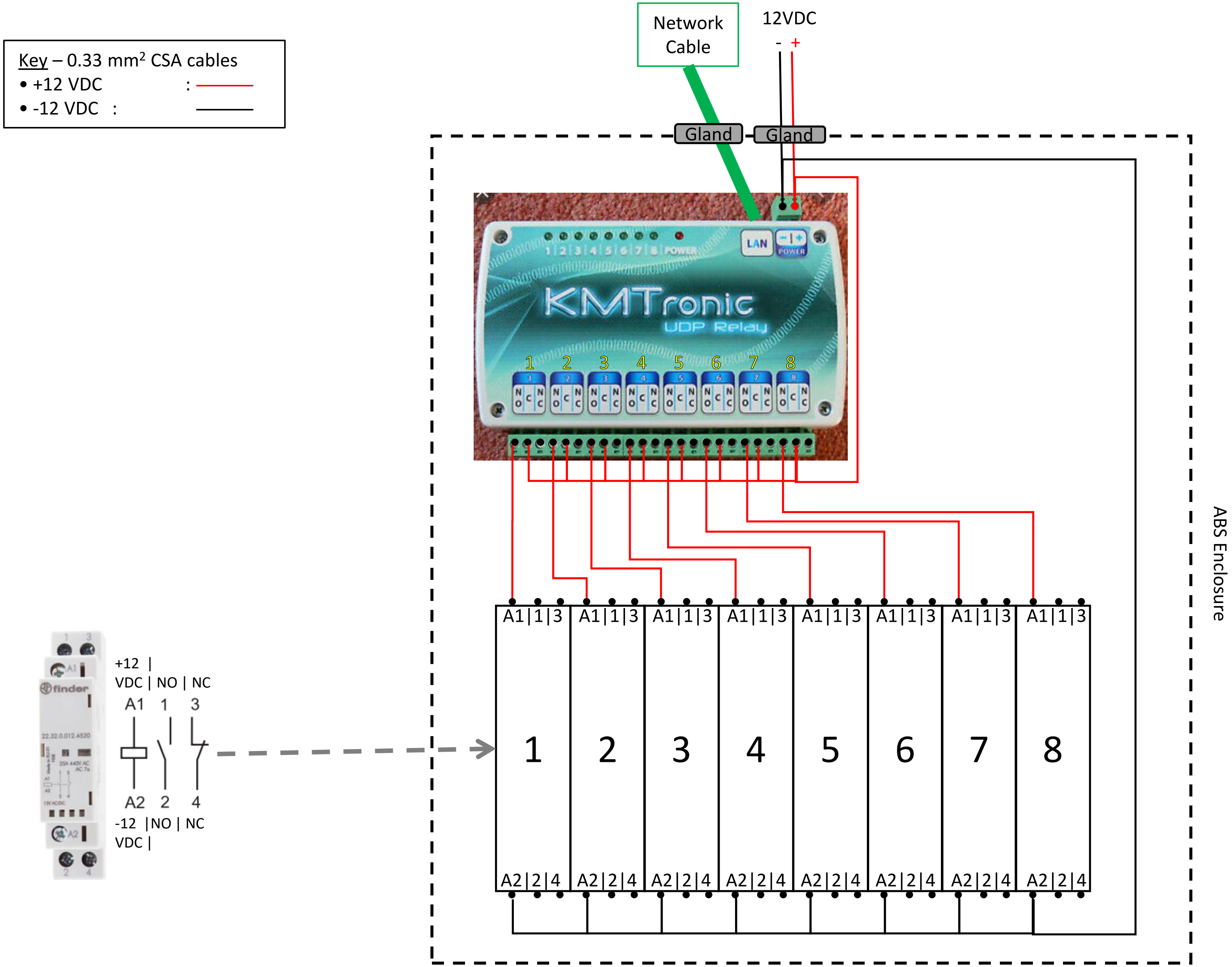

8-Way Contactor Unit – 240VAC connections to control power to equipment (e.g. pumps)

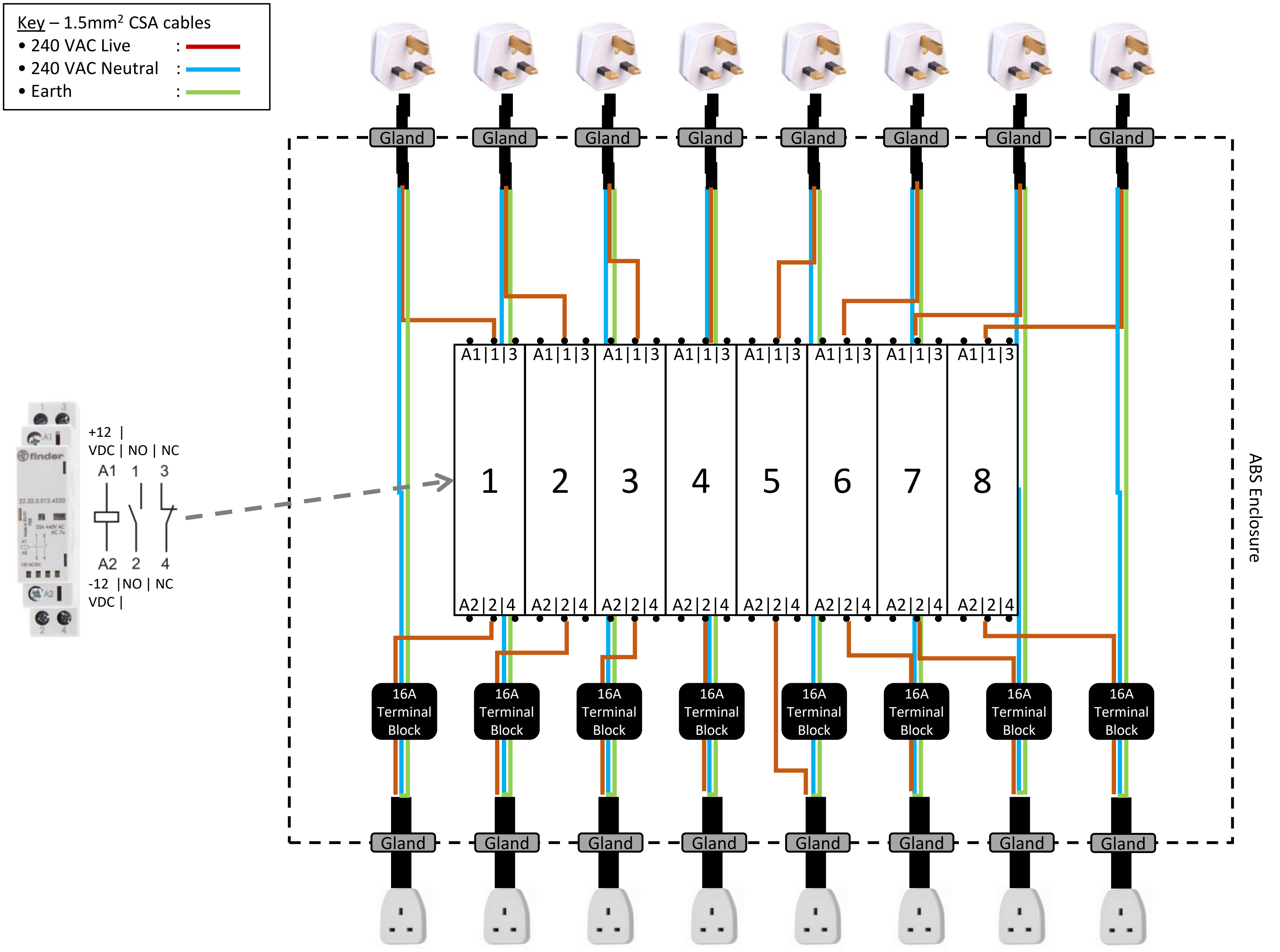

Generator – 32A, 240VAC supply

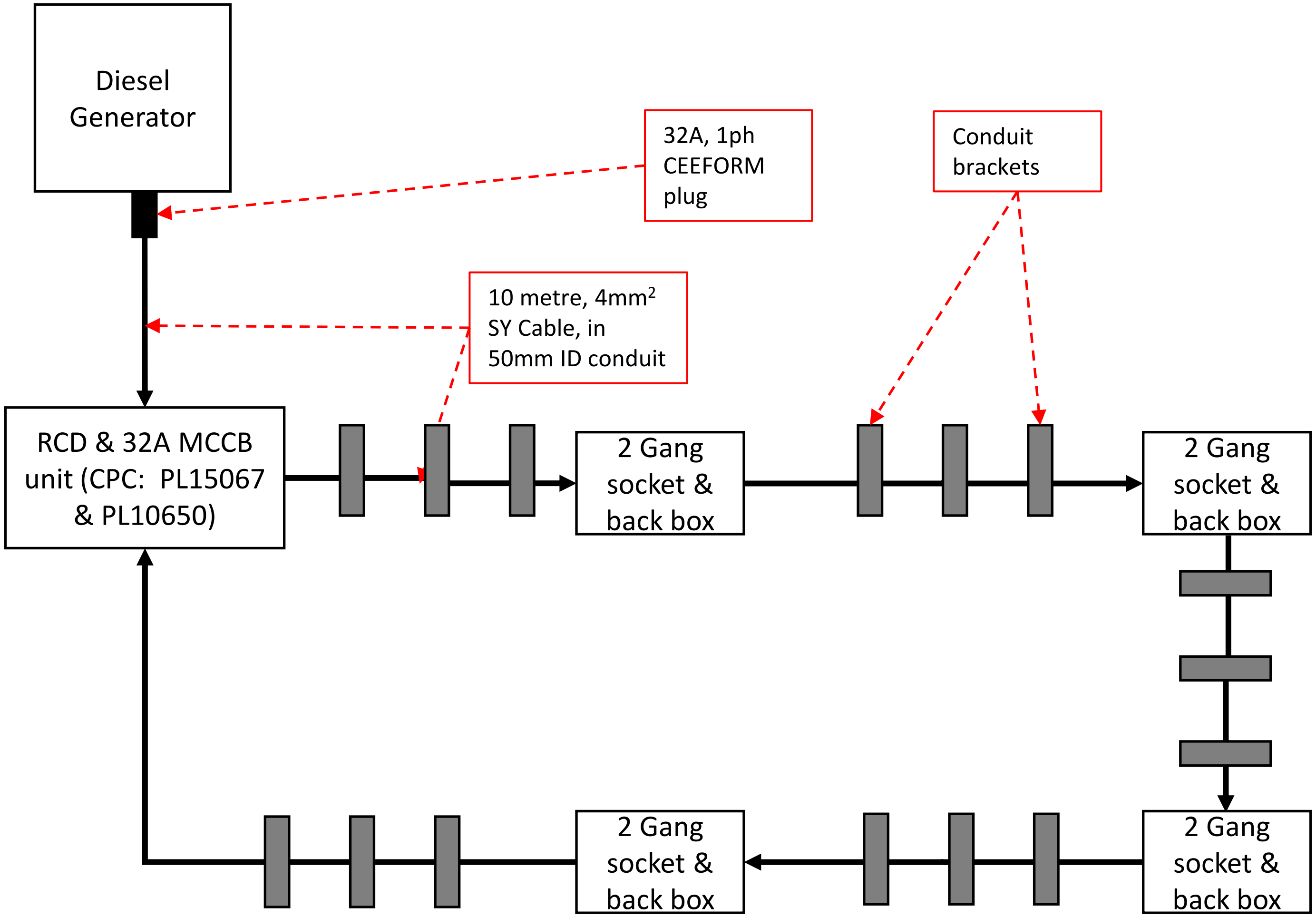

Fumigation Distribution Ring-Side Manifold

PARTS List per manifold

a: 3 off; L 65mm x OD 50mm cut pipe lengths
b: 3 off; 50mm pipe cross piece
c: 8 off; 50mm spigot to 25mm socket adapter
d: 8 off; 25mm OD pipe to hose barb adapter
e: 1 off; 50mm OD pipe to hose barb adapter
f: 1 off; 50mm pipe T piece

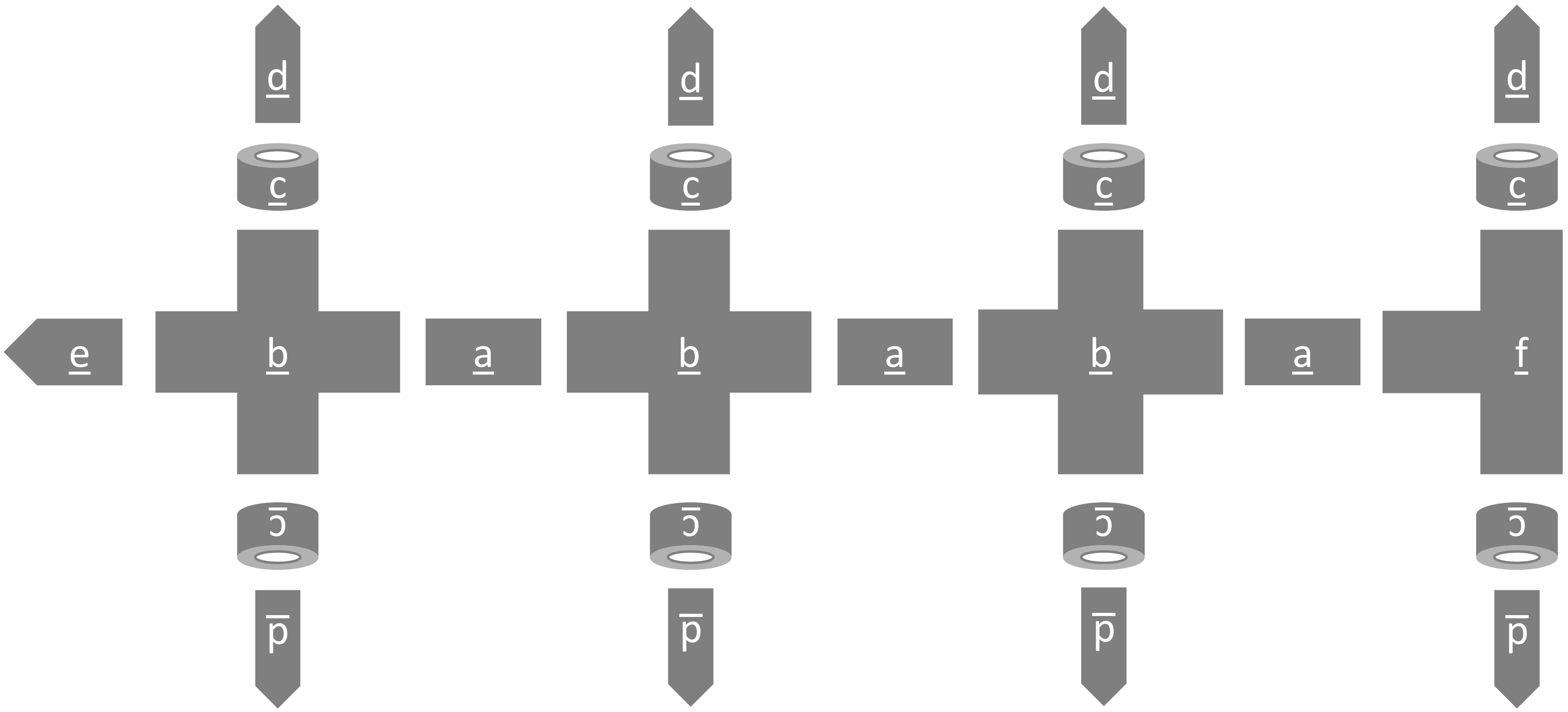

Supplement: Supplementary file 3 [file mmc3.pdf]
