# Supplementary material for: A novel free-air diesel and ozone enrichment (FADOE) research platform
Source: MethodsX. 2024 Feb 27;12:102635. doi: 10.1016/j.mex.2024.102635 (PMC10918276; doi:10.1016/j.mex.2024.102635)
Supplement: Supplementary file 4 [file mmc4.pdf]

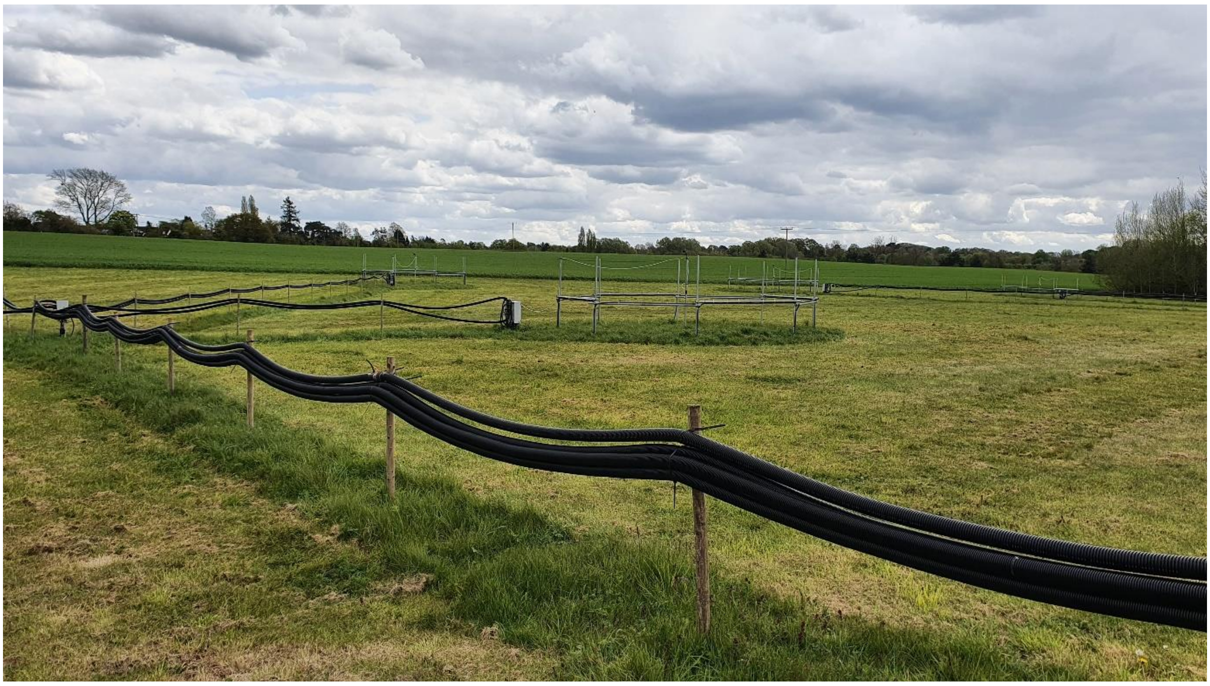
Pollutant/power delivery to ring-side release manifolds

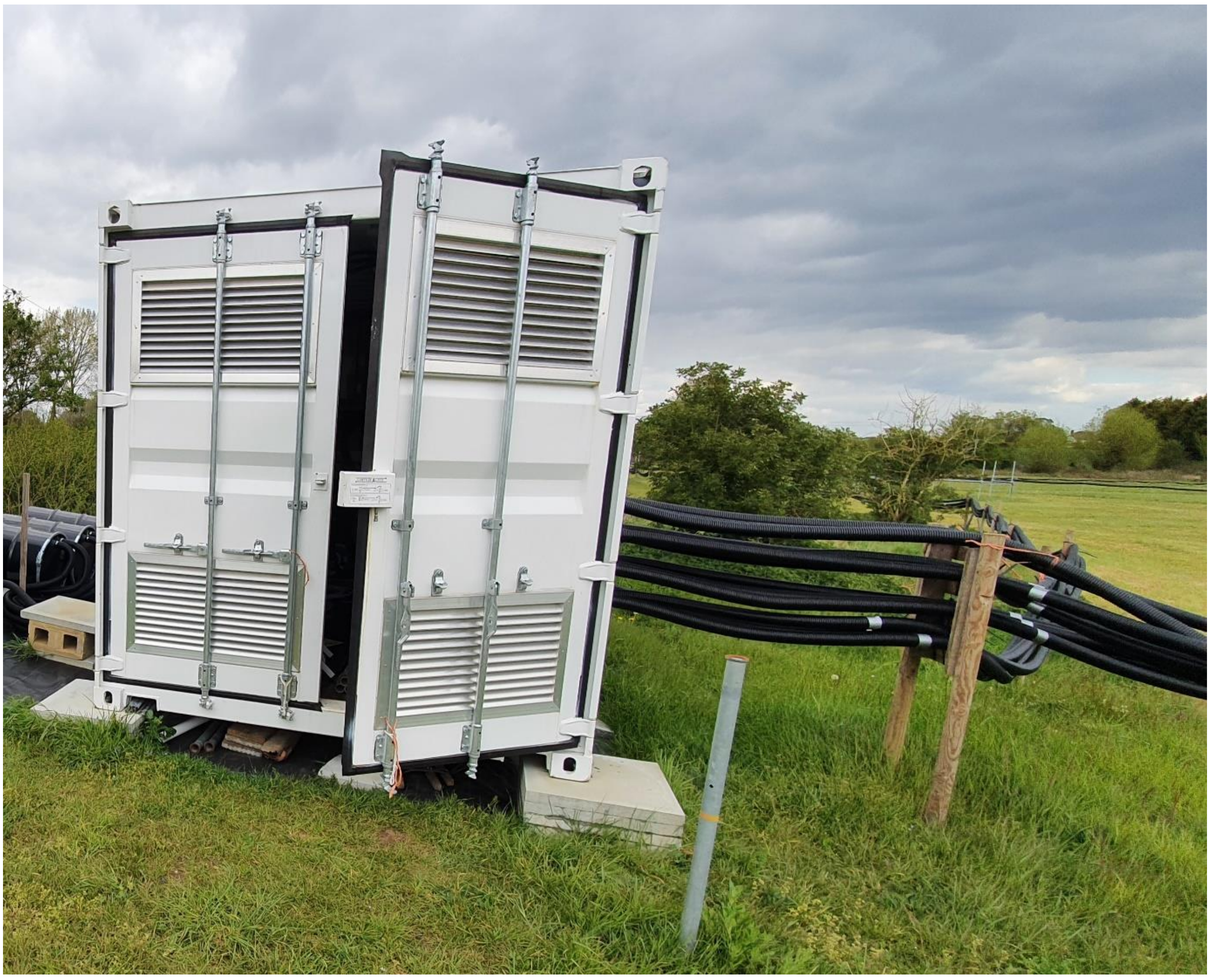
Control Unit Entrance

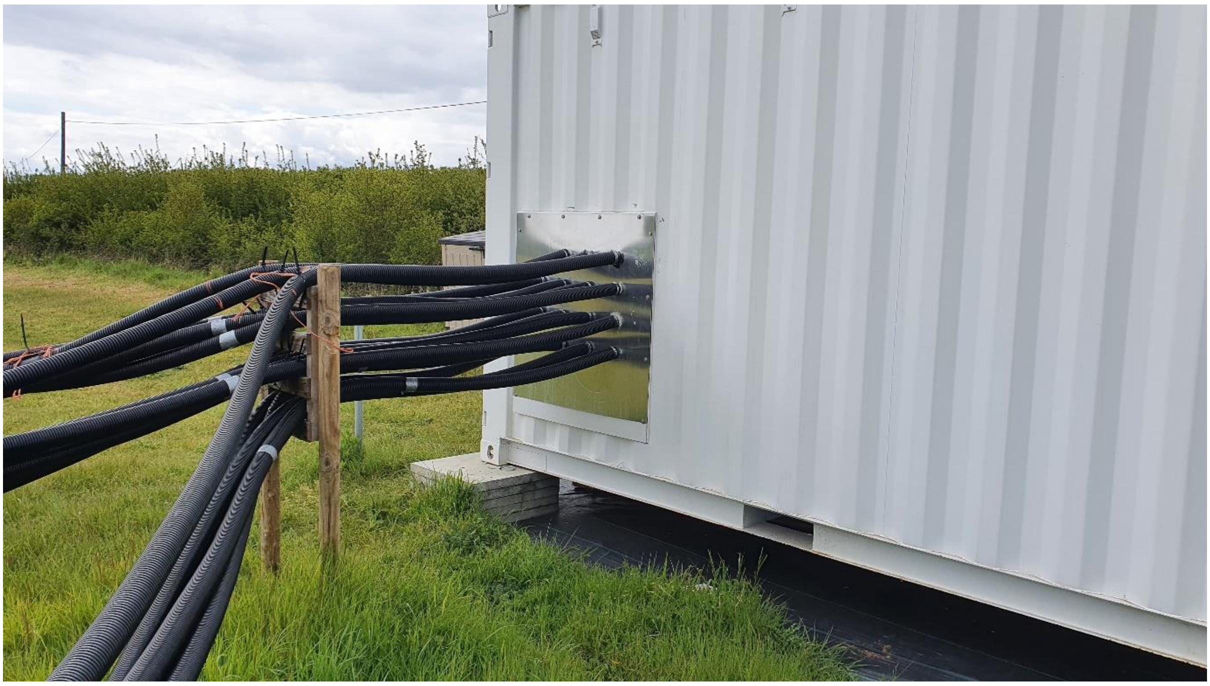
Pollutant/power delivery from Control Unit

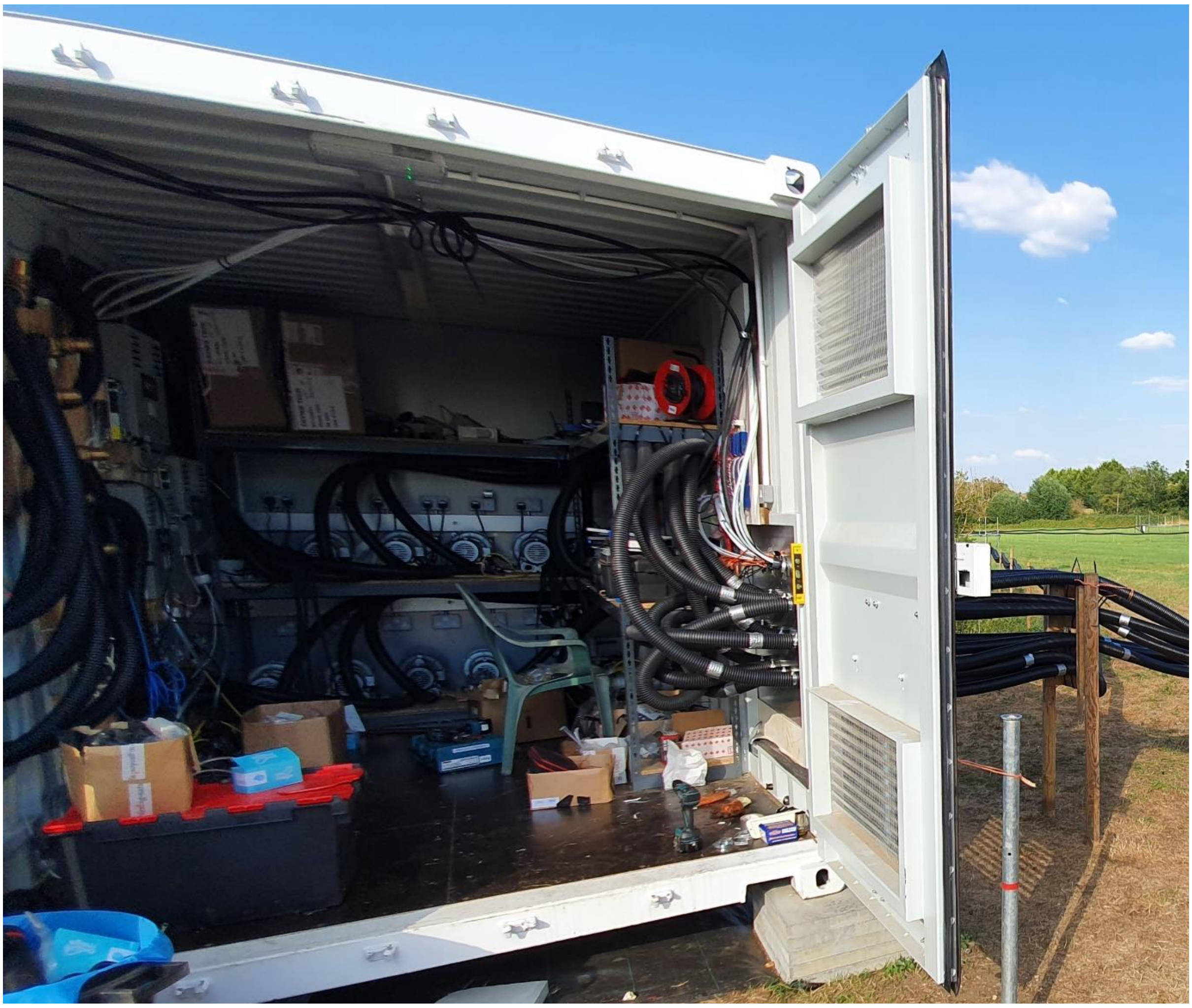
Control Unit Internal

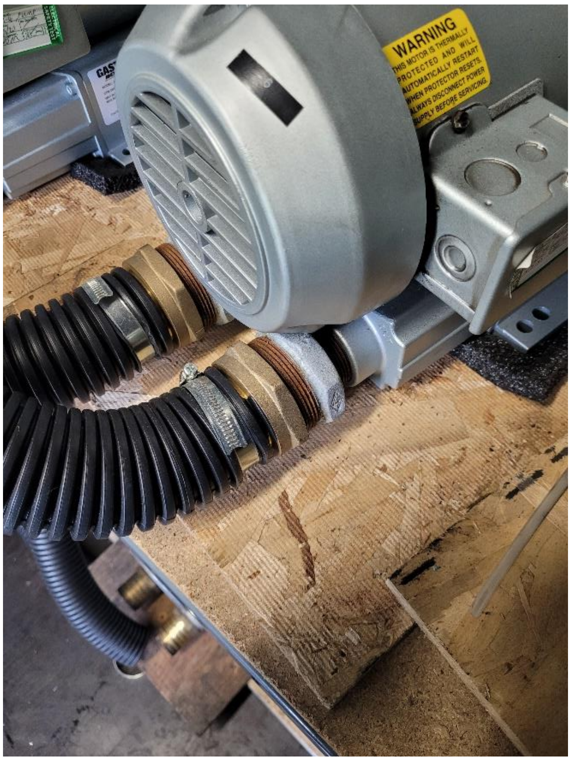

Gast delivery pump

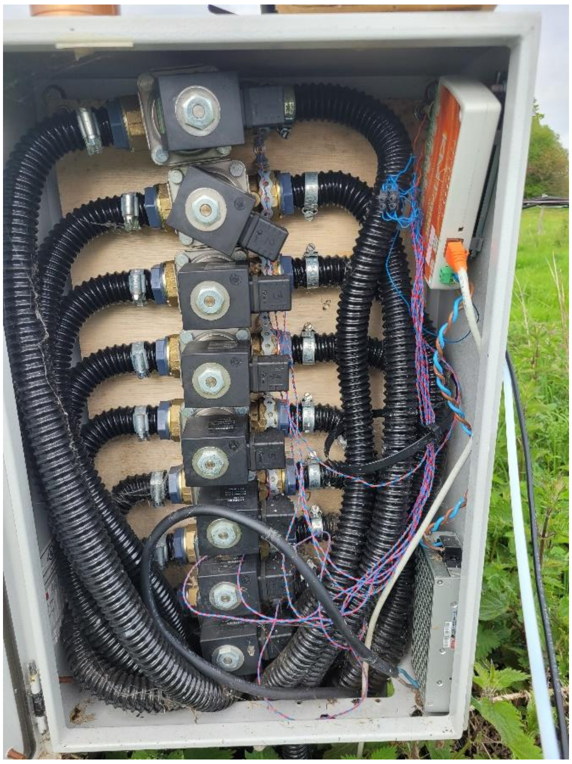
Ring-side release manifold

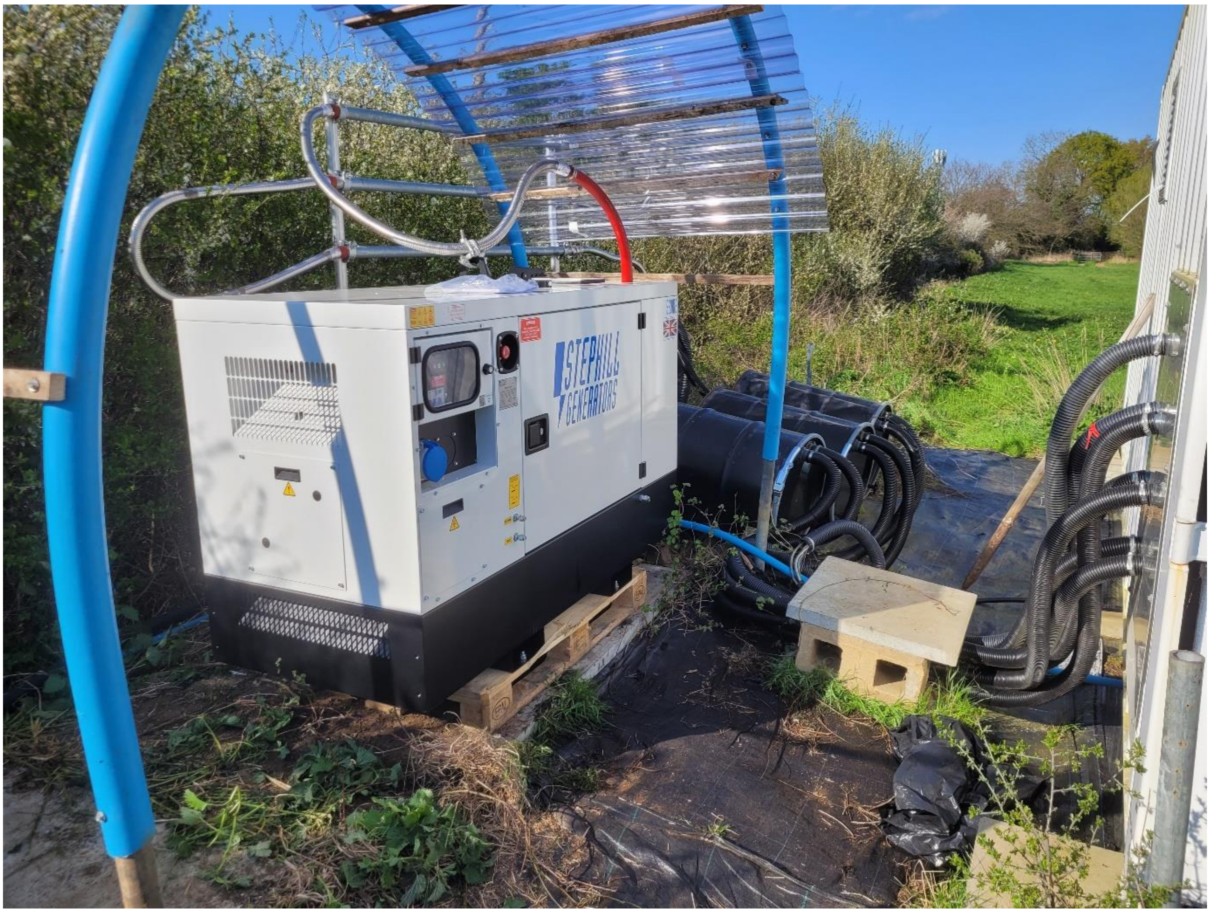

Diesel generator, heat dissipation pipes, mixing barrels

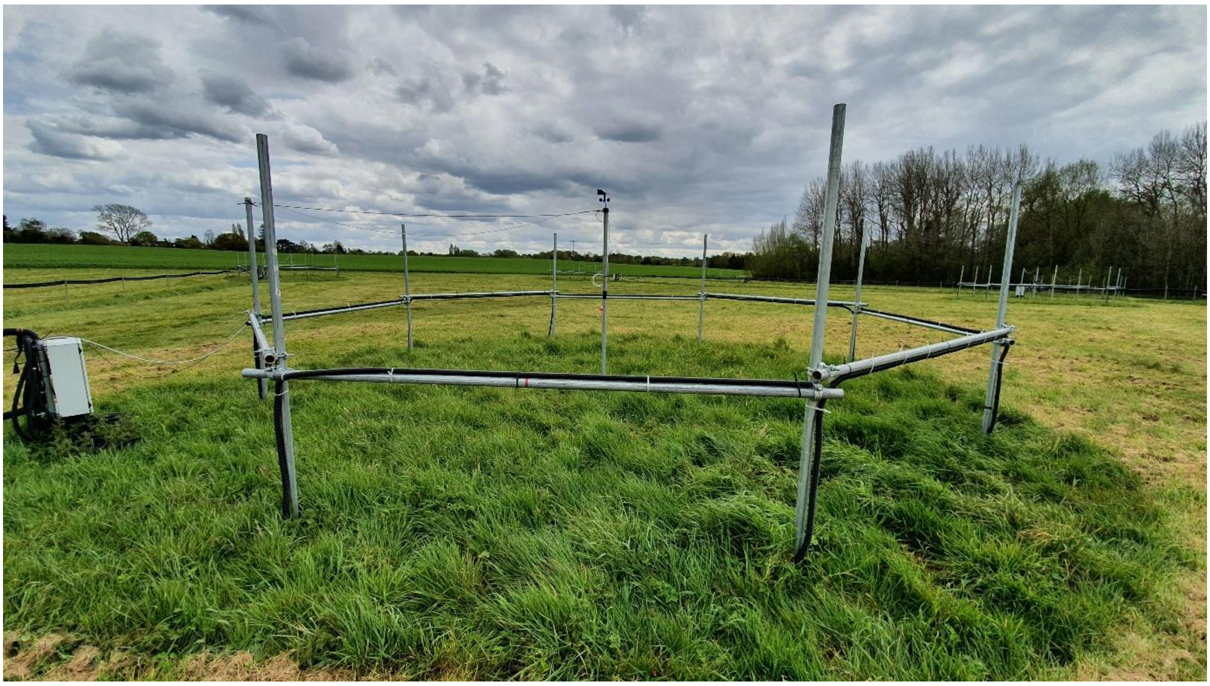

Free-Air Diesel and Ozone Enrichment (FADOE) ring with central sample line and anemometer
